# Niclosamide as a chemical probe for analyzing SARS-CoV-2 modulation of host cell lipid metabolism

**DOI:** 10.3389/fmicb.2023.1251065

**Published:** 2023-10-11

**Authors:** Timothy J. Garrett, Heather Coatsworth, Iqbal Mahmud, Timothy Hamerly, Caroline J. Stephenson, Jasmine B. Ayers, Hoda S. Yazd, Megan R. Miller, John A. Lednicky, Rhoel R. Dinglasan

**Affiliations:** ^1^Department of Pathology, Immunology, and Laboratory Medicine, College of Medicine, University of Florida, Gainesville, FL, United States; ^2^Southeast Center for Integrated Metabolomics, Clinical and Translational Science Institute, University of Florida, Gainesville, FL, United States; ^3^Emerging Pathogens Institute, University of Florida, Gainesville, FL, United States; ^4^Department of Infectious Diseases and Immunology, College of Veterinary Medicine, University of Florida, Gainesville, FL, United States; ^5^Department of Environmental and Global Health, College of Public Health and Health Professions, University of Florida, Gainesville, FL, United States; ^6^Department of Chemistry, University of Florida, Gainesville, FL, United States

**Keywords:** lipidomics, autophagy, lipophagy, COVID-19, RNA sequencing, metabolism, antiviral

## Abstract

**Introduction:**

SARS-CoV-2 subverts host cell processes to facilitate rapid replication and dissemination, and this leads to pathological inflammation.

**Methods:**

We used niclosamide (NIC), a poorly soluble anti-helminth drug identified initially for repurposed treatment of COVID-19, which activates the cells’ autophagic and lipophagic processes as a chemical probe to determine if it can modulate the host cell’s total lipid profile that would otherwise be either amplified or reduced during SARS-CoV-2 infection.

**Results:**

Through parallel lipidomic and transcriptomic analyses we observed massive reorganization of lipid profiles of SARS-CoV-2 infected Vero E6 cells, especially with triglycerides, which were elevated early during virus replication, but decreased thereafter, as well as plasmalogens, which were elevated at later timepoints during virus replication, but were also elevated under normal cell growth. These findings suggested a complex interplay of lipid profile reorganization involving plasmalogen metabolism. We also observed that NIC treatment of both low and high viral loads does not affect virus entry. Instead, NIC treatment reduced the abundance of plasmalogens, diacylglycerides, and ceramides, which we found elevated during virus infection in the absence of NIC, resulting in a significant reduction in the production of infectious virions. Unexpectedly, at higher viral loads, NIC treatment also resulted in elevated triglyceride levels, and induced significant changes in phospholipid metabolism.

**Discussion:**

We posit that future screens of approved or new partner drugs should prioritize compounds that effectively counter SARS-CoV-2 subversion of lipid metabolism, thereby reducing virus replication, egress, and the subsequent regulation of key lipid mediators of pathological inflammation.

## 1. Introduction

The pandemic spread of severe acute respiratory syndrome coronavirus 2 (SARS-CoV-2) has resulted in >1.1 M deaths in the United States alone and > 770 M cases and > 6.9 M deaths worldwide (Dong et al., 2020). In 2020, there was a rapid global effort to study SARS-CoV-2 infection kinetics *in vitro* to identify pathways that are involved in entry, replication, and egress of the virus as new targets that can be treated with existing drugs (Vincent et al., 2005; Liu et al., 2020; Wang et al., 2020; Zhou et al., 2020). However, the worldwide effort since then has been fraught with study inconsistencies ranging from the mammalian host cell used for infection studies, to overall study design., This has made it difficult to identify common host cell pathways critical to virus biology that can be targeted with available compounds. We have an opportunity now to determine how best to identify and evaluate the utility of repurposed drugs during a rapidly evolving pandemic response to global virus threats, such as SARS-CoV-2.

Critical steps in virus entry and egress involve interactions with key host cellular organelles and processes, most especially those involved in autophagy (Rubinsztein et al., 2012; Prabhakara et al., 2021; Chen and Zhang, 2022). Autophagy is a conserved, homeostatic cellular process involving the lysosomal degradation pathway and autophagic degradation of intracellular lipid droplets (lipophagy), as a function of lipid metabolism (Rubinsztein et al., 2012). Indeed, several viruses have been shown to modulate and be affected by rates of lipid droplet metabolism (lipophagy) during infection (Rubinsztein et al., 2012; Chen and Zhang, 2022). As such, it is reasonable to focus on understanding how virus infection affects autophagy to identify new or repurposed drugs that can support interim public health responses to pandemic viruses until vaccines are available.

Chloroquine (CQ) and hydroxychloroquine (HCQ), which are indicated for treating malaria, have been shown to also have antiviral activity against SARS-CoV-2, putatively through their actions on autophagy (Vincent et al., 2005; Dyall et al., 2014; Mauthe et al., 2018; Liu et al., 2020; Wang et al., 2020). However, the direct and rapid translation of this finding to clinical studies during the pandemic was either equivocal at best, or disastrous (Axfors et al., 2021). Although no studies to date have specifically and comprehensively explored the underlying antiviral mechanism of action (MOA) for these two drugs, they nonetheless moved quickly to COVID-19 clinical studies. CQ and HCQ are 4-aminoquinolines (4-AQ). Another human-safe 4-AQ drug, amodiaquine (AQ), has also been shown to have antiviral activity against SARS-CoV-1, Middle East respiratory syndrome coronavirus (MERS-CoV) (Vincent et al., 2005; Dyall et al., 2014), as well as Ebola virus (Mauthe et al., 2018), suggesting that there are common MOAs underpinning broad 4-AQ antiviral activity. These 4-AQ compounds were initially, considered as candidate partner drugs that can be used along with remdesivir (Wang et al., 2020) for the treatment of COVID-19 (Goldman et al., 2021). However, the exact nature of the cellular pathways and processes that are targeted by these 4-AQs resulting in reductions in virus propagation remains unknown. Direct protein interaction studies with CQ suggest broad pleiotropic effects on the cell including the inhibition of autophagosome-lysosome fusion and autophagic flux (Mauthe et al., 2018; Gordon et al., 2020; Wang et al., 2020), which would be problematic at higher doses of the drug used in clinical trials (Borba et al., 2020). Importantly, to date, the use of such drugs for COVID-19 or any virus treatment is discouraged as evidence for their efficacy is not well supported.

Niclosamide (NIC), Another anti-parasitic drug, suppresses MERS-CoV propagation by inhibiting autophagosome-lysosome fusion through the Beclin1 (Bec1, autophagy regulator/antiapoptotic protein)-SKP2 (S-phase kinase-associated protein 2) pathway (Gassen et al., 2019). NIC is a salicylanilide and is an orally administered anti-helminthic drug used in human and veterinary medicine. Akin to the 4-AQs, it also appears to have functional effects beyond the Bec1-SKP2 axis in a cell. As result, despite its broad anti-infective properties *in vitro*, NIC also suffers from poor translational potential to the clinic for COVID-19.

Herein, we explored lipophagy during SARS-CoV-2 infection. Lipophagy is a selective form of autophagy that is influenced by autophagic flux and an important process in lipid metabolism and homeostasis. Lipophagy has been directly linked to flavivirus infection processes in human cells (Zhang et al., 2018; Qin et al., 2022), but a direct link to its involvement has not been fully explored. We profiled the host cell lipidome following SARS-CoV-2 infection and used NIC specifically as a chemical probe to evaluate its effect on the virus-induced lipidomic profile. We hypothesized that NIC exerts pleiotropic functional activities on a host cell that result in the perturbation of two intimately associated cellular pathways: autophagy and lipid metabolism. These two processes are dysregulated at critical SARS-CoV-2 life cycle checkpoints during virus infection (Stukalov et al., 2021), and treatment with NIC is hypothesized to reverse this dysregulation by altering host cell lipid metabolism. Recently, a multi-omics analysis of clinical samples from COVID-19 patients also described a marked change in lipidomics profiles that correlated with disease severity (Overmyer et al., 2021), but this approach could not identify the mechanism at the cellular level. By examining more closely its global effects on host cells we also sought to uncouple NIC’s reported antiviral MOA via the Bec1-SKP2 pathway from intrinsic cytotoxicity (Gassen et al., 2019), which clouds its potential utility as a repurposed drug (Wu et al., 2004; Xu et al., 2020; Prabhakara et al., 2021). We identified and characterized autophagic or lipophagic pathways (and wider linked cellular networks) that are targeted by NIC in the presence and absence of a SARS-CoV-2 infection and used the lipid signatures induced by this anti-parasitic compound to detect pathways that are predicted to either lead to direct antiviral effects or to cellular dysregulation and cell death. Finally, we discuss the utility of this approach in informing the selection of repurposed drugs that may have more clinical benefit, better bioavailability, and lesser cytotoxicity to support future pandemic virus responses.

## 2. Results

### 2.1. Lipidomic and transcriptomic profile of Vero E6 cell cultures

**Figure 1:**
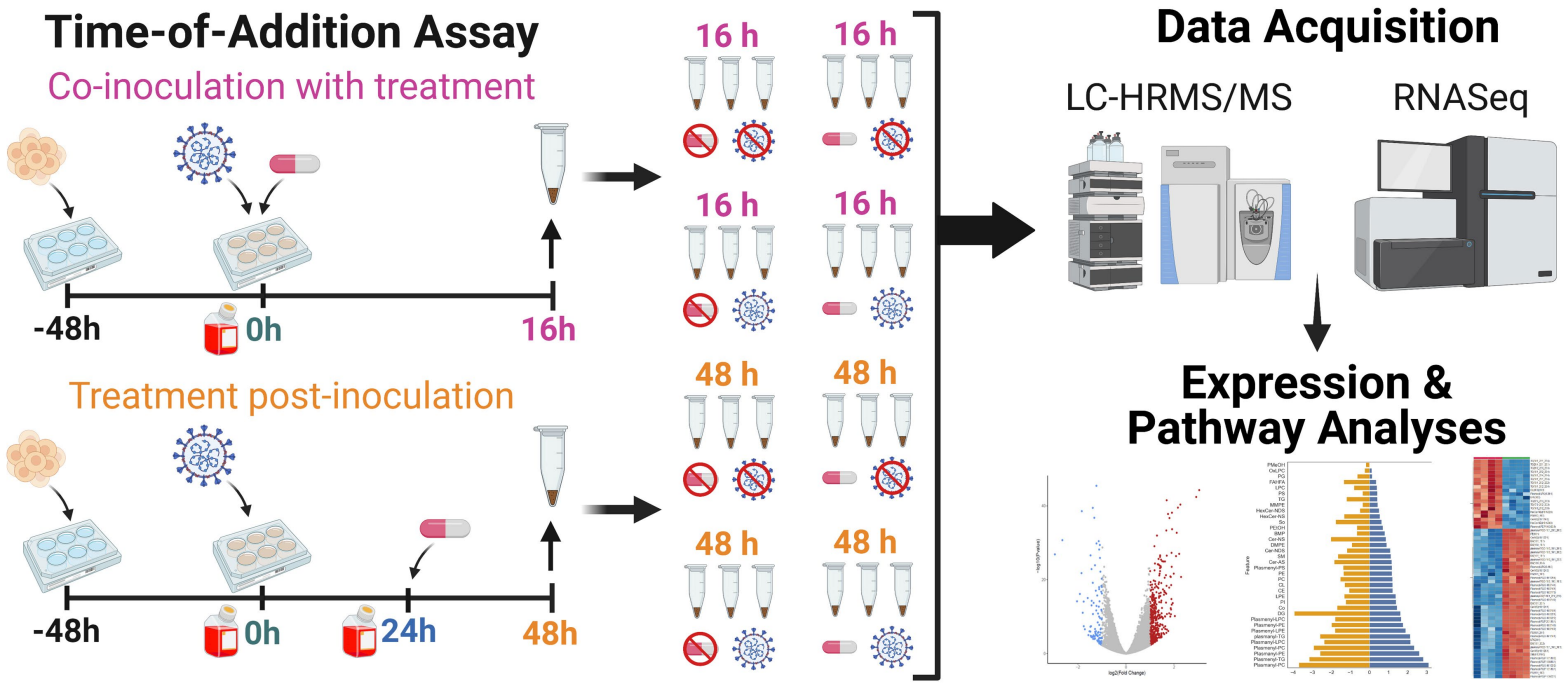
Workflow for lipidomic and transcriptomic profiling of Vero E6 cells after SARS-CoV-2 infection with and without niclosamide treatment. We used a time-of-addition assay experimental design to (i) capture the lipidomic profile of SARS-CoV-2 infected Vero E6 cells, and (ii) explore the effect of niclosamide on the lipidomic profile of Vero E6 cells when added in the absence of infection, with SARS-CoV-2 virus, or at 24 h post-infection. Each replicate experimental condition (*n* = 3) was processed for LC-HRMS/MS or RNASeq analyses. Samples were seeded 48 h prior to the start of the experiment (*t* = 0 h). For all samples, media was changed at the start of the experiment (*t* = 0 h) and infected with virus (for infected sample groups). Sample collection is denoted by an up arrow and tube above the timeline, media changes are denoted by red bottles, addition of virus and DMSO/drug are denoted as well. Separate samples for each condition were collected for LC-HRMS/MS and RNAseq analysis, respectively. Created with BioRender.com.

**Figure 2:**
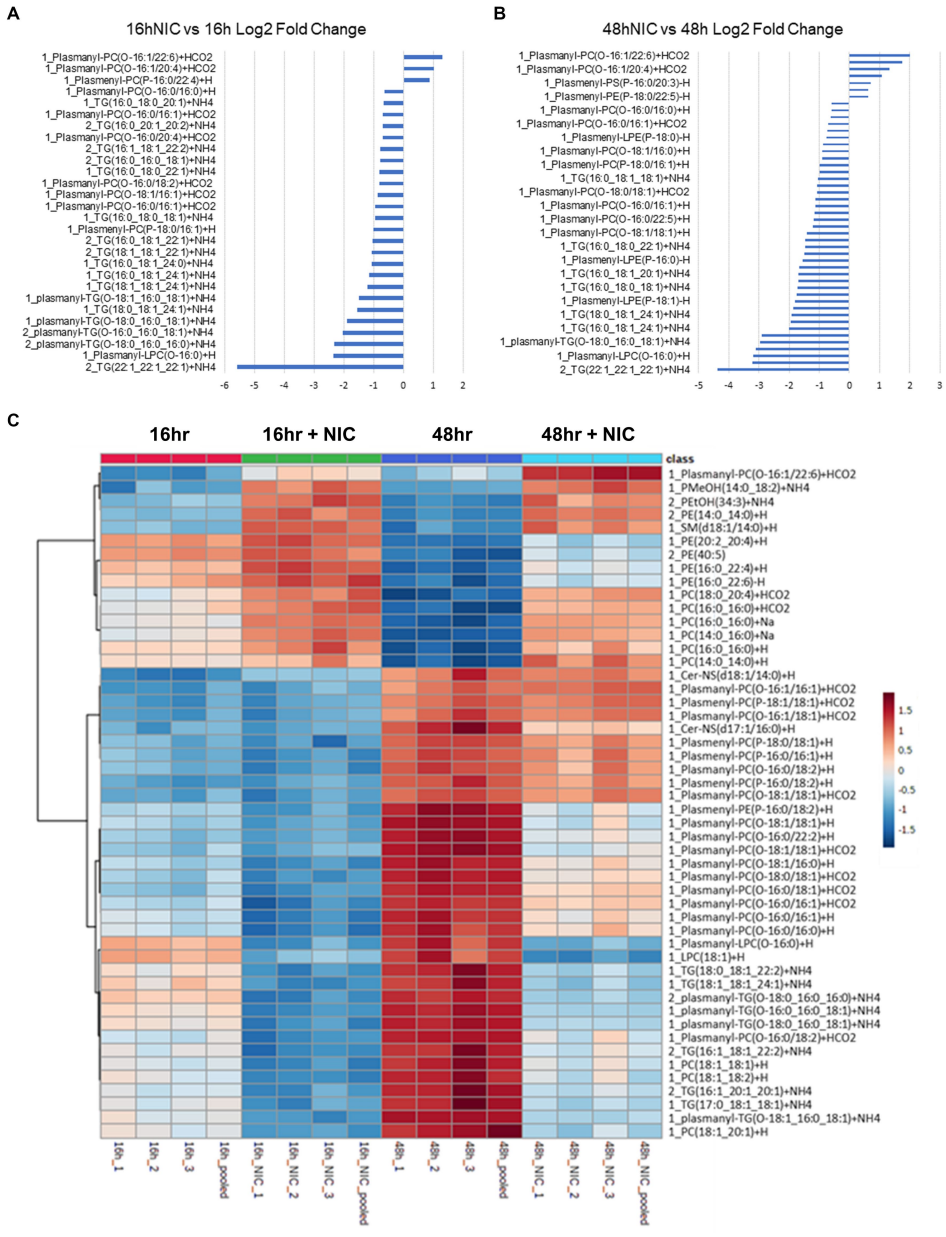
Niclosamide modulates lipid metabolism in Vero E6 cells in the absence of SARS-CoV-2 infection. (A,B) Bar graphs combining the log P-value (colored in blue) and the Log2 fold change (colored in orange) for total lipid expression at 16h vs 16h NIC (A) and 48h vs 48h NIC (B), data is sorted by low to high fold change. (C) Heatmap clustering of the top 50 lipids across all classes measured in Vero E6 cells showing the differential lipid regulation from 16h to 48h. NIC treatment increased several phosphatidylcholines (PC) and decreased several triglycerides (TG) comparing 16hr to 48hr. TG and PC Plasmalogens were increased at 16hr and 48hr without NIC but were decreased at 16hr and 48hr following NIC treatment.

To achieve a comprehensive multiomics analysis of lipophagy and lipid metabolism, we first examined Vero E6 cell responses using a time-of-drug-addition assay approach (Aoki-Utsubo et al., 2018). This approach examines the cell response in the absence of infection, and with or without NIC treatment to establish a baseline for both the 16 h and 48 h time points of the virus intracellular life cycle (Figure 1). Lipidomic analysis showed a decrease in the expression of plasmalogens (ether-linked lipids) and triglycerides (TG) at the later timepoint with NIC treatment (Figures 2A,B). Transcriptomic analysis did not identify any significantly differentially expressed (DE) genes over time in our Vero E6 culture alone (0 h vs. 16 h, 0 h vs. 48 h, 16 h vs. 48 h) (See Supplementary Table S1), suggesting that while the cellular background of Vero E6 cells during these different time periods may be changing as a function of culture, the age of the cell culture did not confound other comparisons made in this study.

### 2.2. NIC treatment distinctly impacts ether lipids in the absence of infection

**Figure 3:**
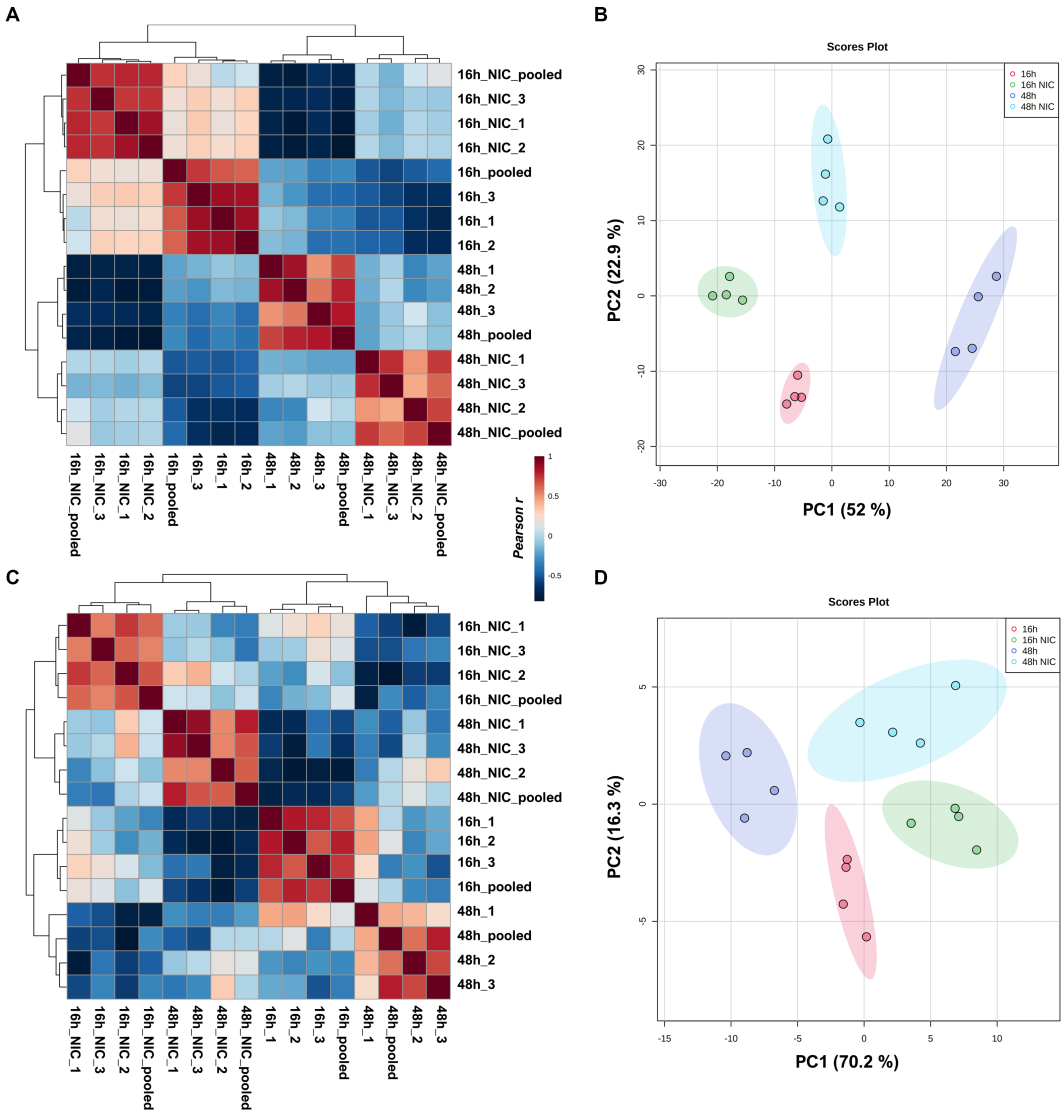
Clustering analysis of virus free Vero E6 cell cultures comparing early growth (16 h) and late growth (48 h) with and without NIC. Pearson correlation and PCA including all lipids identified **(A,B)**. Pearson correlation and PCA including only TG lipids detected **(C,D)**. Clustering was evident in all cases, but correlation with all lipid expressions was primarily based on time points while correlation using only TG expression was from NIC treatment.

In total, we identified 520 differentially expressed lipids across all samples, with distinct profiles between DMSO (vehicle) control and NIC treated cells (See Supplementary Table S2). We observed a profound reduction across both time points in the lipid profiles for plasmalogens and TG, and this effect was more pronounced at the 48 h time point (Figures 2A-C, and see Supplementary Table S3). It is clear from our results that NIC alters the plasmalogen and TG content early as we observed a significant decrease at 16 h + NIC compared to 16 h without NIC and this host lipid alteration continues to a greater extent at 48 h + NIC compared to 48 h without NIC (Figure 2B). In the absence of NIC treatment, plasmalogens (plasmanyl-PE, −TG, and -PC as well as plasmenyl-LPC, -PE, -LPE, -PC and -TG) were found to be differentially abundant at 48 h as compared to 16 h, which is expected given the cell growth during this time frame (Nahapetian et al., 1986; Nardacci et al., 2021) (Figures 2A,C, and see Supplementary Table S4). However, at the 48 h time point (24 h post drug treatment) NIC reduced the abundance (>2 log2 fold-change) of HexCer-NDS, while in parallel, increased the abundance (>2 log2 fold-change) of plasmenyl- and plasmanyl-TG, PI, plasmanyl-PE and -PC, as well as Bis (monoacylglycerol)phosphates (BMPs) (Figure 2B). Upon treatment with NIC, it appears that this lipid pathway is disrupted significantly. The correlation matrix across the groups with and without NIC showed a distinct clustering when evaluating all lipids (Figure 3A) as well as when looking only at TG lipids (Figure 3C). In addition, principal components analysis (PCA) revealed clear clustering based on lipid profiles between early (16 h) and later (48 h) time points with and without NIC using total lipids (Figure 3B) and only TG lipids (Figure 3D).

### 2.3. SARS-CoV-2 infection alters the host cell lipidome

**Figure 4:**
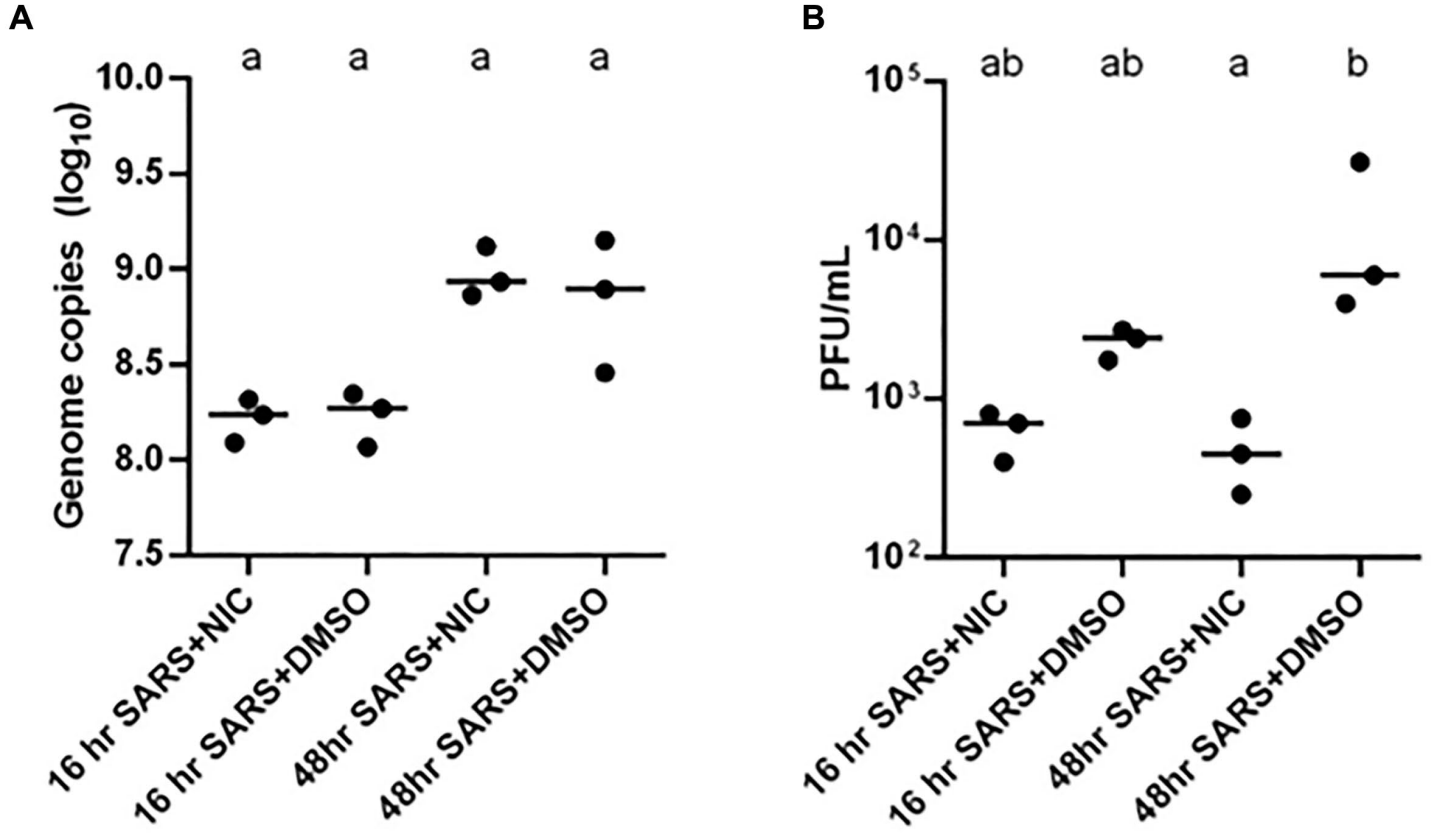
Impact of Niclosamide (NIC) treatment on SARS-CoV-2 genome copy and infectious virion production. **(A)** The addition of NIC did not impact the log_10_ genome copy number of SARS-CoV-2 treatments at 16 or 48 h. There was a significantly higher number of genome copies in the 48 h treatments than the 16 h SARS-CoV-2 and NIC treatment. **(B)** The addition of NIC significantly reduced the infectious virion production (PFU/mL) of SARS-CoV-2 at 48 h; although this trend was also present at 16 h, it was not statistically significant. Statistically significant differences between treatment conditions (three biological replicates represented by each dot) were determined using a two-way non-parametric ANOVA with Dunn’s post-hoc test was performed in GraphPad Prism v.6.0 and assessed at an *α* = 0.05. Treatments without a common letter were found to be statistically significant.

The mechanism by which SARS-CoV-2 enters host cells and systematically alters the cellular environment remains one of the most compelling areas of study to support the development of antiviral interventions (V’kovski et al., 2021). Evidence suggests that SARS-CoV-2 subverts pre-existing cellular lipids and lipid- signaling mechanisms for entry, intracellular trafficking, and egress (Mazzon and Mercer, 2014; Theken et al., 2021). To investigate the role of lipids in SARS-CoV-2 during the infection cycle in host cells, we profiled the global lipidome from Vero E6 cells at 16 h and 48 h post-infection with SARS-CoV-2. To increase relevance, we used an infectious dose that mimics natural exposure (i.e., low multiplicity of infection, MOI) through aerosols in enclosed settings experienced by the majority of the population (Lednicky et al., 2020; Coleman et al., 2022; Vass et al., 2022), and then subsequently assessed for consistency of the observations at a higher infectious dose or MOI. The correlation matrix across the groups studied showed a distinct clustering in SARS-CoV-2 infected cells as well as clear clustering with virus and NIC treatment (Supplementary Figures S1A,B, respectively), discussed below. In addition, PCA revealed clear clustering based on lipid profiles between early (16 h) and late (48 h) timepoints post viral infection (Supplementary Figure S1C). Furthermore, PCAs also showed clustering with NIC treatment of virus infected cells at early (16 h) and late timepoints (48 h) (Supplementary Figures S1D,E, respectively). Measurement of supernatant SARS-CoV-2 genome copy with RT-qPCR revealed a significantly higher quantity of released progeny genomes at 48 h than 16 h, indicating that more viral egress is occurring at the 48 h timepoint, which is consistent with literature (Kavaliauskiene et al., 2014; Ju et al., 2021) (Figure 4).

**Figure 5:**
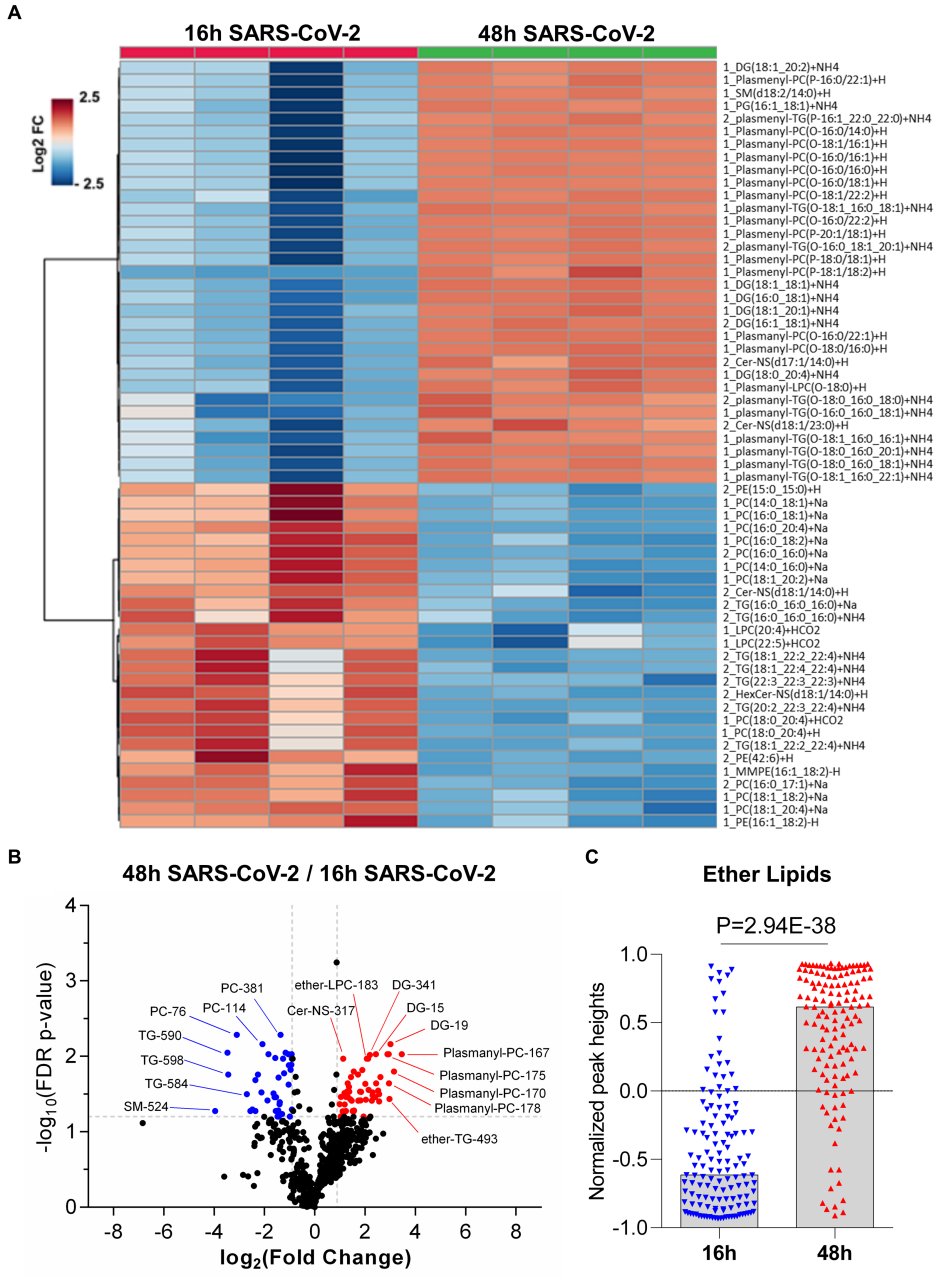
Global lipidomics analysis in SARS-CoV-2 infected Vero E6 cells at 16 h and 48 h. **(A)** Hierarchical cluster heatmap analysis depicting the major affected lipid clustering between 16 h and 48 h infection. **(B)** Volcano plot showing the differential lipid abundance with SARS-CoV-2 infection between 16 h and 48 h. The primary significantly upregulated lipids include plasmalogens, diglycerides and triglycerides. **(C)** Bar graph showing the total abundance of ether lipids between the time points. Lipid molecule abbreviations (shown in panel **B**): PC-76 [1_PC (16,0_20:4) + Na], PC-114 [1_PC (18,1_20:4) + Na | 1_PC (16,0_22:5) + Na], PC-381 [2_PC (16,0_17:1) + Na | 2_PC (15,0_18:1) + Na], TG-584 [2_TG (18,1_22:4_22:4) + NH4], TG-590 [2_TG (20,2_22:3_22:4) + NH4], TG-598 [2_TG (22,3_22:3_22:3) + NH4], SM-524 [2_SM (d17:1/20:3) + H], Cer-NS-317 [2_Cer-NS (d17:1/14:0) + H], DG-15 [1_DG (16,0_18:1) + NH4 | 1_DG (16,1_18:0) + NH4], DG-19 [1_DG (18,1_18,1) + NH4 | 1_DG (16,0_20,2) + NH4 | 1_DG (16,1_20,1) + NH4 | 1_DG (18,0_18,2) + NH4], DG-341 [2_DG (16:1_18:1) + NH4 | 1_DG (16,1_18,1) + NH4 | 1_DG (16,0_18,2) + NH4 | 1_DG (14,0_20,2) + NH4], Ether-LPC-183 [1_Plasmanyl-LPC (O-18:0) + H], Ether-TG-493 [2_plasmanyl-TG (O-16:1_20:0_20:0) + NH4 | 2_plasmenyl-TG (P-16:0_20:0_20:0) + NH4], Plasmanyl-PC-167 [1_Plasmanyl-PC (O-18:0/16:0) + H], Plasmanyl-PC-170 [1_Plasmanyl-PC (O-16:0/22:2) + H], Plasmanyl-PC-175 [1_Plasmanyl-PC (O-16:0/22:1) + H], and Plasmanyl-PC-178 [1_Plasmanyl-PC (O-18:1/22:2) + H | 1_Plasmenyl-PC (P-18:0/22:2) + H].

As SARS-CoV-2 infection caused perturbations in the global lipid profiles (Figure 5), we next sought to characterize the modulations of different lipid classes between early (16 h) vs. late (48 h) timepoints post virus infection. In total, we identified 720 lipids from 34 classes (See Supplementary Table S5) in SARS-CoV-2 infected host cells covering all major lipid classes (Figure 5A). Phosphatidylcholine (PC), phosphatidylethanolamine (PE), plasmalogens, and TG represented the most frequent lipid classes identified, which is expected given their abundance in cell membranes and lipid droplets (Figure 5).

The repertoire of lipid classes that partitioned to early steps in virus infection (16 h) and later stages of increased virus replication and egress (48 h) are shown in Figure 5. Hierarchical clustering analysis of the differentially regulated lipids (FC > 1.5, adjusted value of *p* <0.05) revealed specific lipid classes were associated with early or late events during productive virus infection (see Supplementary Table S5). TGs, cholesterol esters (CE), and hexosylceramides (HexCer) were elevated at early stages of infection (16 h) (Figure 5 and see Supplementary Table S5). Lipids that contained longer chain fatty acids with higher degrees of unsaturation including TGs (4–9 double bonds (db)), PC (4–5 db), CL (5–6 db), PE (1–6 db), and PS (1–4 db) were elevated in early (16 h) viral infection (Figure 5A, see Supplementary Table S5), a trend consistent with what was described in SARS-CoV-2 infected golden Syrian hamsters (Rizvi et al., 2022). In contrast, saturated fatty acids (SFA), monounsaturated fatty acids (MUFA) and long chain fatty acids (LCFA) were found to be significantly downregulated in early (16 h) viral infection but elevated at 48 h post viral infection (See Supplementary Table S6), consistent with previous reports (Koyuncu et al., 2013; Nguyen et al., 2018).

We also found that plasmalogens including plasmanyl-TG, plasmenyl-TG, plasmanyl-LPC, plasmanyl-PC, plasmenyl-PC, and plasmenyl-PE, as well as diacylglycerides (DG) were elevated at 48 h post virus infection (Figures 5A,B, see Supplementary Table S6). We measured the relative abundance of the total ether-linked lipids and observed a highly significant elevation (*p*-value = 2.94E-38) of total ether-linked lipids at 48 h post virus infection (Figure 5C). A comparative extracted ion chromatogram of plasmanyl-PC (O-18:0/16:0) + H, as an example of a specific ether lipid (Supplementary Figure S4), conveys the clear difference in profiles between the two time points tested. However, it should be noted that we also observed a similar increase in plasmalogens during normal (uninfected) cell growth comparing 16 h vs. 48 h (Figure 2C), suggesting that SARS-CoV-2 infection is not disrupting all ether-linked lipid regulation in Vero E6 cells.

Altogether, we identified changes in lipids that may be related to a cellular response to compensate for virus energy utilization, viral particle formation from lipid droplets (Dias et al., 2020), vesicle transport and autophagosome formation, noting a significant increase in plasmalogens, sphingolipids (SM and CerNS), glycerophospholipids (PI, PS, and PG), lysophospholipids (LPE) and glycerolipids (DG) and a significant decrease in TG and CE lipids at 48 h (Figure 5); which was not observed under normal cell growth. We observed a differential regulation of lipids at 48 h post-infection vs. 16 h post-infection. Of note, we observed significant increases in PG, PS, and PI, but not a significant change in PC or PE phospholipids. This differential regulation of phospholipids could suggest formation of membranes for the viral envelope as most mammalian cells are high in PC and PE phospholipids (Deng and Angelova, 2021).

### 2.4. Effect of NIC on SARS-CoV-2 infected cells

NIC was previously found to suppress MERS-CoV propagation by enhancing autophagic flux through the Beclin1-SKP2 pathway (Gassen et al., 2019), and as such, NIC was proposed to be a potent candidate drug for repurposing in the treatment of SARS and COVID-19 (Xu et al., 2020; Prabhakara et al., 2021). The influence of SARS-CoV-2 infection on Vero E6 cell metabolism, and inhibition of autophagic flux encouraged us to assess how NIC can potentially modulate SARS-CoV-2 infection-induced changes to host cell lipid metabolism. We analyzed the genome copy and infectious virion production of SARS-CoV-2 with and without NIC and determined that in our hands, NIC does not reduce genome copy number (Figure 4A) but reduces infectious SARS-CoV-2 virion production at 16 h and 48 h (Figure 4B). Although the reduction was more modest at 16 h as compared to the log-fold reduction observed at 48 h, both were statistically significant. Importantly, treatment with 5 μM NIC alone did not significantly change the number of viable cells compared to vehicle controls at either timepoint, as measured by the cell counting kit 8 assay (Supplementary Figures S2A,B). However, cells infected with SARS-CoV-2 and then treated with NIC or NIC treatment alone had significantly reduced viability compared to cells treated with virus plus DMSO vehicle at 16 h (Supplementary Figure S2C). At 48 h, virus plus NIC and virus plus vehicle conditions were equivalently viable, but both were significantly less viable than the vehicle control alone (Supplementary Figure S2D). This indicates that despite NIC reducing the quantity of infectious virus particles produced by the cells, it does not rescue the cells from the cytopathic effect of virus infection.

**Figure 6:**
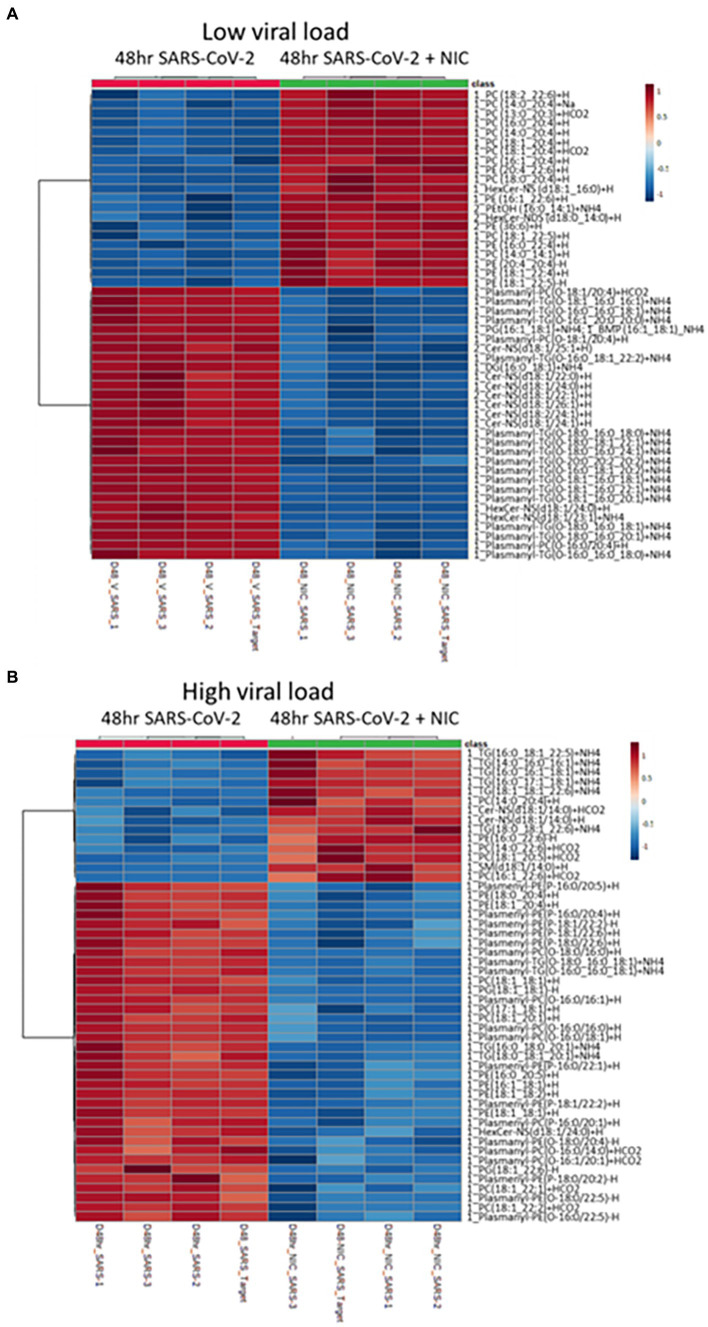
Impact of Niclosamide (NIC) treatment on lipid production during low and high viral load. **(A)** Heatmap of the top 50 lipids with a low viral load showing an increase in phospholipids (PL) with a high amount of unsaturation under NIC treatment. **(B)** Heatmap of the top 50 lipids with a high viral load showing an increase in both triglycerides (TG) and phospholipids having a high degree of unsaturation, but a decrease in TG and PLs with lower degrees of unsaturation under NIC treatment. We observed a decrease in plasmalogens at both high and low viral load.

Having established a baseline effect of NIC on lipid metabolism in Vero E6 cells, we next sought to use NIC as a probe to manipulate host cell lipid metabolism during SARS-CoV-2 infection using two different infectious doses, at a low and a high MOI. The infectious dose is an important factor in assessing the MOA for a drug in the treatment of a virus infection, especially if it is more relevant to actual transmission dynamics. A lower MOI represents what can be expected from a natural, non-clinical aerosol exposure that leads to downstream infection (Lednicky et al., 2020; Coleman et al., 2022), and the higher MOI, would represent what clinicians and first-responders would be exposed to in a virus-rich ambulatory or indoor hospital setting, with virus exposure resulting from a high rate of expulsion of a combination of virus in large droplets and aerosols (Lednicky et al., 2020; Cheng et al., 2021; Ju et al., 2021; Coleman et al., 2022; Vass et al., 2022). We globally profiled lipids from cells infected with SARS-CoV-2 and then treated with DMSO and cells infected with SARS-CoV-2 and treated with or without NIC at 48 h (Figure 6; Supplementary Table S6) using our UHPLC-HRMS approach. A PCA on the lipidomic data identified a clear separation between NIC treated and untreated SARS-CoV-2 infected samples (Supplementary Figures S1C–E). We observed that the treatment of Vero E6 cells with NIC for 24 h starting at 24 h after SARS-CoV-2 infection (48 h) robustly impacted the global lipid profile as compared to NIC treatment beginning simultaneously with SARS-CoV-2 infection (Figures 6A,B), suggesting that effective antiviral activity of NIC is dependent on time of addition. At low viral load (Figure 6A), we observed a reduction in plasmalogens, DG, CerNS, and HexCer-NS with NIC treatment, which would have otherwise increased during SARS-CoV-2 infection (or under normal cell growth conditions) at the 48 h time point (Figure 6A, also Figure 5). We also observed in parallel, an increase in phospholipids (PC and PE) at 48 h that had higher degrees of unsaturation including 20:4, 22:5, and 22:6 containing fatty acids; a change that we did not observe during virus infection (no NIC) at this time point. We then compared these results with the lipidomic changes associated with NIC treatment at a higher MOI after 48 h (Figure 6B). We observed a similar reduction in plasmalogens, but in contrast to the lower MOI condition, we noted an unexpected increase in TGs. Furthermore, although we also identified a small number of phospholipids (PC and PE) with higher degrees of unsaturation that increased in abundance, we also observed a reduction in phospholipids that contain lower degrees of un-saturation (less than 2 double bonds).

### 2.5. Transcriptional profiling captures the broader cellular impact of virus infection and treatment with NIC

**Figure 7:**
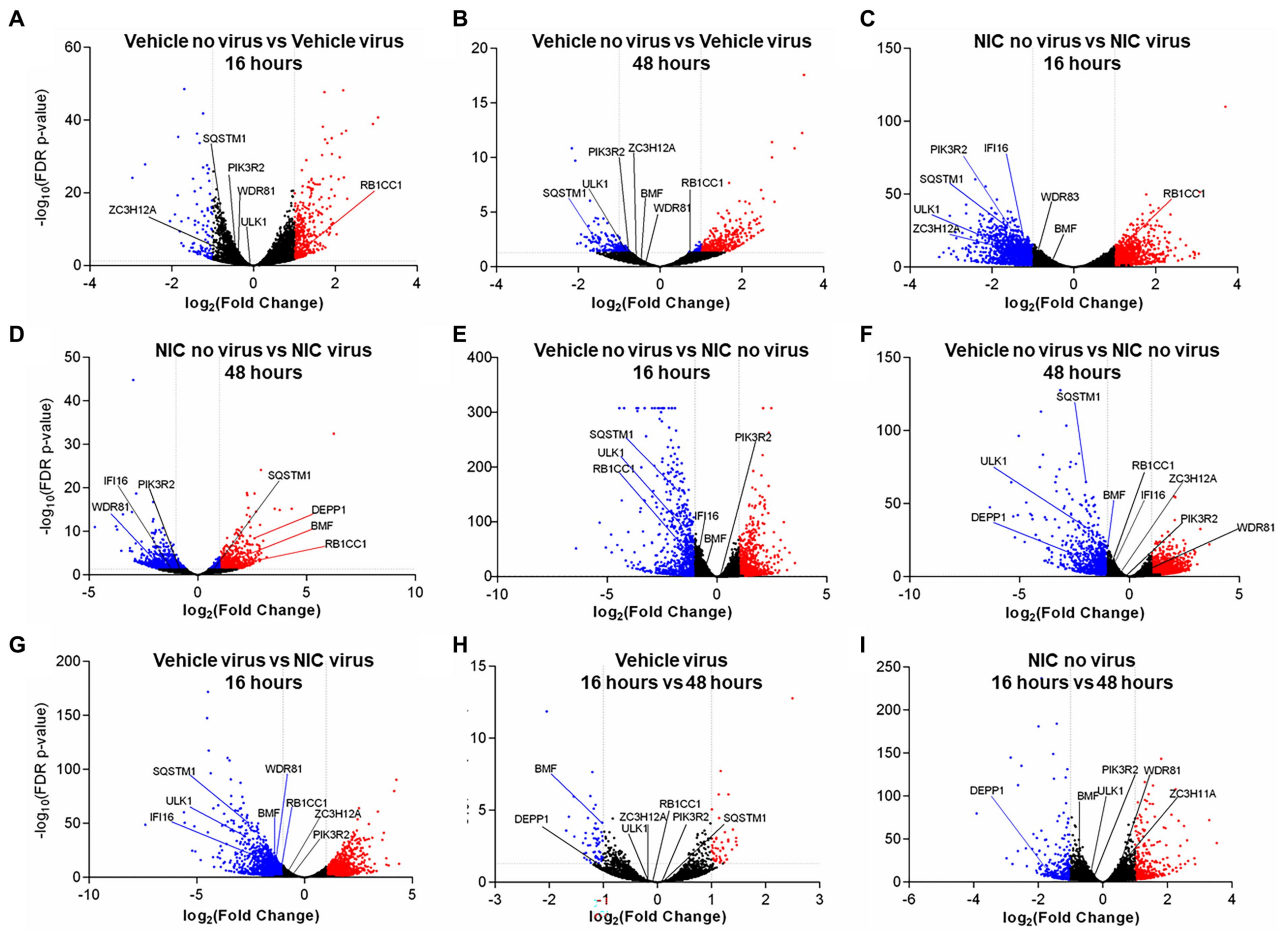
Significantly differentially expressed genes across SARS-CoV-2 and Niclosamide conditions. Genes were designated as significantly expressed using DESeq2 when their Bonferroni adjusted p-value was ≤0.05, and the |log2 (Fold Change)| was ≥1. Each dot represents an individual gene. Blue dots are significantly downregulated genes, red dots are significantly upregulated genes, and grey dots are not significantly differentially expressed genes. Comparisons analyzing the effect of virus by comparing no virus and virus conditions include: **(A)** DMSO treated no virus vs. DMSO treated, virus samples at 16 h, **(B)** DMSO treated no virus vs. DMSO treated virus samples at 48 h, **(C)** Niclosamide-treated no virus vs. niclosamide-treated virus samples at 16 h, and **(D)** Niclosamide-treated no virus vs. niclosamide treated virus samples at 48 h. Comparisons analyzing the effect of NIC by comparing no NIC and NIC conditions include: **(E)** DMSO-treated no virus vs. niclosamide treated no virus samples at 16 h, **(F)** DMSO-treated no virus vs. niclosamide treated no virus samples at 48 h, and **(G)** DMSO-treated virus vs. niclosamide treated virus samples at 16 h. Lastly, comparisons analyzing the effect of time by comparing 16 h and 48 h conditions are: **(H)** DMSO-treated virus at 16 h vs. 48 h, and **(I)** Niclosamide-treated, no virus samples at 16 h vs. 48 h.

We assessed if virus-induced changes in lipidomic profiles corresponded to canonical transcriptional regulation of genes that are known to be involved in lipid metabolism, autophagy, phosphorylation, and vesicle transport. Furthermore, we explored if treatment with NIC alters this profile, and whether time of NIC addition alters the course of infection. To do this, we used RNASeq to capture the global cellular gene expression of (i) Vero E6 cells over time (effect of time), (ii) Vero E6 cells during cellular SARS-CoV-2 infection (effect of virus), and (iii) following treatment with NIC (effect of drug) (Figure 7).

We did not observe any DE genes between cells treated with SARS-CoV-2 and NIC at 16 h vs. 48 h (Supplementary Table S7). We did, however, observe differences with NIC treated cells at 16 h vs. 48 h (860 DE genes, 474 up-regulated and 386 down-regulated at 48 h) (Supplementary Table S8), as well as SARS-CoV-2 infected cells at 16 h vs. 48 h (109 DE genes, 48 up-regulated and 61 down-regulated at 48 h) (Supplementary Tables S7, S8).

There were significant transcriptomic differences noted at 16 h and 48 h with NIC treatment (Figures 7E,F), and at 48 h with NIC treatment and SARS-CoV-2 infection (Supplementary Table S8; Figure 7D; Supplementary Figure S3). NIC treatment alone (compared to no NIC treatments) induced 2,447 DE genes at 16 h (1,301 up-regulated, 1,146 down-regulated), and 2,846 genes at 48 h (1,707 up-regulated, 1,139 down-regulated), while viral infection and NIC treatment (compared to treatments with virus but without NIC) resulted in 3,617 DE genes at 16 h (1,698 up-regulated, 1,919 down-regulated), although no DE genes were noted by 48 h (Figures 7E–G; Supplementary Table S8; Supplementary Figure S3). Overrepresented GO terms with NIC treatment related to autophagosome assembly, regulation of autophagy, intracellular lipid metabolism, lipid localization and lipid homeostasis (Supplementary Figure S3). Similar genes were noted associated with the effect of NIC as the effect of virus. RB1CC1 was up-regulated at 16 h with virus infection (Figure 7A) but was down-regulated upon NIC treatment (Figure 7E) at 16 h. Conversely, BMF was down-regulated with NIC at 48 h, but was up-regulated upon NIC and virus treatment at 48 h. Other genes that were down-regulated with viral infection (GOLGA2, IFI16, WDR81, HK2, SQSTM1, ULK1) remained down-regulated with NIC treatment (with and without virus).

SARS-CoV-2 infection resulted in a significant number of DE genes across all our comparison groups (Supplementary Table S8; Figure 7; Supplementary Figure S3). Viral infection (as compared to treatments without virus) resulted in 585 DE genes at 16 h (474 up-regulated, 111 down-regulated), and 562 DE genes at 48 h (433 up-regulated, 129 down-regulated) (Supplementary Table S8). SARS-CoV-2 and NIC conditions resulted in more DE genes than SARS-CoV-2 infection alone; NIC and viral infection (compared to treatments with NIC but without virus) resulted in 3,083 DE genes (1,301 up-regulated, 1,782 down-regulated) at 16 h, and 1,595 DE genes (706 up-regulated, 889 down-regulated) at 48 h (Supplementary Table S9). Overrepresented GO terms associated in both up- and down-regulated DE datasets contained several terms associated with lipid phosphate metabolism, lipid transport, autophagy, and lysosome activities. Terms related to phosphatidylinositol, phosphatidylethanolamine and phosphatidylglycerol were especially prevalent, mirroring observed changes in the lipidomics data (Supplementary Figure S3). At 16 h, viral infection induced an up-regulation of GABARAPL1, MAP1LC3A, PIK3C3, USP30, and TBK1 (also up-regulated at 48 h), and a down-regulation of STK11, LARP1, ZC3H12A, TFEB, TICAM1, GOLGA2, PIK3R2, IFI16, ULK1, WDR81, SQSTM1 (also down-regulated at 48 h), and HK2. As per viral infection, NIC and virus treated cells (at 48 h) also saw an up-regulation of GABARAPL1, MAP1LC3A, USP30, BMF, and TBK1; WDR81 was also down-regulated in the NIC and virus treatment at 48 h.

## 3. Discussion

A virus subjugates host lipid molecules as a vehicle for entry, intracellular transport, virus replication, assembly of infectious viral particles, and egress (Miyanari et al., 2007). Ether lipids have been identified as an important lipid class for efficient membrane trafficking, endocytosis, transcytosis, and internalization of particles (Thai et al., 2001). Deficiency of ether lipids in several model systems affects plasma membrane function, as well as structural changes in the ER and Golgi cisternae (Bazill and Dexter, 1990; Gorgas et al., 2006). SARS-CoV-2 infection has demonstrated a diverse range of COVID-19 disease severities that correlate with increased viral replication (Fajnzylber et al., 2020). However, the role of lipid metabolism or ether lipids in particular during SARS-CoV-2 infection has not heretofore been described. Unlike during the early infection stage at 16 h, where virus load and replication is low, we observed that SARS-CoV-2 infection elevated primarily PC plasmalogens after 48 h of infection, a phase that represents increased virus replication, virus assembly and egress as well as host cell energy utilization (Dimitrov, 2004; Mazzon and Mercer, 2014; Theken et al., 2021). Under normal Vero E6 cell growth, we observed an increase in plasmalogens at 48 h, but the plasmalogens were a mix of TG, PC, and PE. Clearly, follow-up studies focusing entirely on just plasmalogens is needed to further explore their role during virus infection.

We observed that NIC treatment significantly downregulates the ether lipid profile during infection. We also observed that NIC treatment in Vero E6 cells significantly alters the host lipidome along several pathways including a reduction in TG content at both 16 h and 48 h suggesting a disruption of lipid droplet formation and reducing the availability of TGs for viral replication (Figure 2A). NIC has previously been tested as an anti-obesity drug, and a study using it associated with a high fat diet showed a decrease in total TGs, thus our results confirm that NIC directs a clear modification of host lipid levels (Al-Gareeb et al., 2017). NIC treatment alone also affects several other major lipid species, including PE, which may stimulate the autophagy process and inhibit ether lipid elevation during viral propagation. However, NIC has dual activity in cells and is capable of influencing autophagic processes through canonical and non-canonical pathways (Liu et al., 2019). Whereas in uninfected cells, NIC reduces autophagic flux as well as the global lipid profile (causing a decrease in plasmalogens, TGs and PE lipids), in infected cells, NIC induces autophagy and the subsequent reduction of the ether lipid profile. NIC has been shown to affect the function of lysosomes by preventing the acidification of the vacuole (Jung et al., 2019). This disruption of lysosomal function results in increased autophagic flux similar to the likely primary mechanism of action underlying the broad antiviral activity of chloroquine (Chen and Zhang, 2022). Importantly, we identified that ether-linked lipids were only affected by NIC treatment relative to productive virus infection at 48 h, suggesting that the activity of NIC may be dependent on the cellular state or level of virus burden and virus replication kinetics.

To understand the critical lipids related to autophagy activation, we analyzed the PE levels in NIC treated SARS-CoV-2 infected cells (Figure 6). PE is one of the central lipids that conjugates the factors for induction of autophagy through the formation of the autophagosome (Xie et al., 2020). We observed that the relative abundance of PE was significantly elevated with NIC treatment at 48 h while there was no impact of NIC on PE levels at 16 h, suggesting that NIC enhanced the autophagy machinery upon viral replication to directly affect downstream virus assembly, trafficking, and egress. Different signaling lipids such as PS, PI, and PGs play critical roles in endosome trafficking and maturation (Mazzon and Mercer, 2014) and help in infectious viral particle transport. Our study first identified that NIC treatment significantly reduced the global level of different glycerophospholipids such as PS, PI, and PG at 48 h, but had no impact on early infection at 16 h.

TGs are the major form of lipids enriched in lipid droplets. Lipid droplets are used by cells to store neutral lipids that are utilized for energy needs, but viruses require these lipids for replication and thus hijack the lipid droplets to enable their own growth. In fasting conditions, lipid droplets are utilized for fatty acid oxidation to produce energy as intact TGs are broken down to release fatty acids in a process called lipophagy. Drugs that stimulate TG downregulation usually activate autophagy and lipophagy and may protect a cell from virus infection. In support of this hypothesis, we observed that NIC treatment in the absence of virus lowers TG levels at 48 h. However, we also observed that SARS-CoV-2 infection in the absence of NIC also reduces TG levels at 48 h. The significant decrease in TGs and CEs during virus infection minus drug treatment is likely related to virus utilization of lipid droplets to make viral particles needed for replication. The increase in PC plasmalogens, which are involved in lipid droplet formation is also associated with viral replication. Increased levels have been detected in the serum of patients with ZIKV and other viruses (Mazzon and Mercer, 2014; Deng and Angelova, 2021). However, we also noted that normal host cell growth at 48 h results in an increase in plasmalogen levels but a mixture of PC, PE, and TG. Considering that we observed clear downregulation of TG with NIC alone, control of the host TG content could be a key driver in preventing viral replication. This is an observation that is often missed by proteomics and transcriptomic studies exploring SARS-CoV-2 infection and the mechanism of action of antivirals *in vitro*. Unexpectedly, NIC treatment of a SARS-CoV-2 infection at 48 h, resulted in increased TG levels, indicating that normal cell growth occurring at 48 h plus increased viral replication can overcome the effects of NIC on the lipidomic profile of SARS-CoV-2 infection of Vero E6 cells and suggests that the dose of NIC needs to be adjusted based on viral load.

Bis (monoacylglycerol)phosphates (BMP), identified as a new lipid of endosome-derived extracellular vehicles (EVs), also act as cholesterol transporters in cooperation with other fac-tors and facilitate virus infection and replication (Luquain-Costaz et al., 2020). Interestingly, our study detected three different forms of BMP and two different forms of cholesterol esters (CE) that were all significantly downregulated with NIC treatment. Besides the impact of NIC on different autophagy related lipids, we also investigated the role of NIC on bioactive lipid metabolism and apoptosis. We observed a dichotomous pattern of CerNS and SM lipids, where CerNS decreased after NIC treatment (48hV vs. 48 hV + NIC) while SM were increased, indicative of cell death or cell survival, which may be partly explained by the cell state-dependency of NIC that we have observed in the presence or absence of SARS-CoV-2 infection in Vero E6 cells. Interestingly, we identified a notable reduction of gangliosides detected as HexCer (which are also called glycosphingolipids) upon NIC treatment at 48 h in both uninfected and SARS-CoV-2 infected Vero E6. Gangliosides have been implicated in the induction of autophagic death in mammalian cells (Hwang et al., 2010), suggesting that NIC can increase cell survival at homeostasis as well as under stress/infection conditions. This activity may in part contribute to the low cytotoxicity for this drug for several different cell lines, with the reported CC_50_ = 250 μM (Zhang et al., 2018; Prabhakara et al., 2021). Although gangliosides have also been shown to bind cooperatively with ACE-2 to the SARS-CoV-2 spike protein (Fantini et al., 2021), our experimental design did not include a pretreatment of cells prior to virus infection. The search for highly safe drugs for prophylaxis against COVID-19 remains elusive and controversial, but these data may suggest a role for NIC in future outbreaks given its broad antiviral activities and its control of the host lipidome. Considering the potential link between plasmalogens and the observed cytokine and lipid storms (Dias et al., 2020) in severe COVID-19 patients, NIC may offer to a two-pronged treatment approach, i.e., reduce virus abundance by reducing host lipids necessary for virus production and provide a check on an uncontrolled inflammatory response induced by SARS-CoV-2.

These findings are further supported by the global transcriptome profile that we captured for each of the treatments/conditions, wherein hallmarks of and the induction of autophagy related genes (such as DEPP1 (an autophagy regulator) (Salcher et al., 2017)), several lipases (Wu et al., 2004), as well as genes implicated in the induction of apoptosis and genes that are markers of cell proliferation. Furthermore, GO overrepresentation analyses showed significantly upregulated terms such as death receptor activity, MAP kinase phosphatase activity, tumor necrosis factor-activated receptor activity, and phosphatidylinositol kinase activity (Supplementary Figure S3). To investigate the role NIC has on the reversal of SARS-CoV-2 infection induced autophagy dysregulation, we explored the function of several genes that were differentially expressed. RB1CC1 (RB1 inducible Coiled-Coil 1) along with ULK1 are part of the ULK complex that initiates and regulates autophagy (Zachari and Ganley, 2017). RB1CC1 has also been shown to influence viral infection (Mauthe and Reggiori, 2016), as a depletion of RB1CC1 led to an increase of encephalomyocarditis virus replication. Here, RB1CC1 was up-regulated by SARS-CoV-2 at 16 h; however, was significantly down-regulated with treatment of NIC at 16 h. We also observed down-regulation of ULK1 in NIC treated cells at 16 h. This elucidates the potential role of RB1CC1 and ULK1 in early infection with SARS-CoV-2 and illuminates how NIC may regulate autophagy. Interestingly, BMF was down-regulated at 48 h with NIC, but up-regulated upon NIC treatment of virus infected cells at 48 h. BMF encodes for a Bcl-2-modifying factor that is responsible for apoptotic regulation and has been re-ported as a target facilitating viral evasion (Zamaraev et al., 2020). Additionally, autophagy genes IFI16 (Duan et al., 2011; Wichit et al., 2019; Kim et al., 2020; Jiang et al., 2021), ZC3H12A (Srivastava et al., 2020), SQSTM1 (Wu et al., 2004), WDR81 (Zhu et al., 2021), and PIK3R2 (Weisberg et al., 2020) are associated with viral infection, including SARS-CoV-1 (Wu et al., 2004; Srivastava et al., 2020) and were downregulated in this study when cells were treated with NIC. Taken together, this provides evidence that SARS-CoV-2 infection leads to dysregulation of autophagy, and that NIC acts to reverse this effect.

Several omics studies have made a significant contribution to the identification and understanding of potential antiviral therapies for COVID-19 (Mahmud and Garrett, 2020). Bioactive molecules including PUFA lipids such as oleoylethanolamide (OEA), arachidonic acid (AA), eicosapentaenoic acid (EPA), and docosahexaenoic acid (DHA) are known from existing viral pathogenesis to inactivate enveloped viruses and inhibit pathogen proliferation/replication (Tam et al., 2013; Das, 2020; Ghaffari et al., 2020). Accumulation of lipids including sphingolipids such as ceramides has been shown to negatively affect viral pathogenesis (Young et al., 2013; Soudani et al., 2019). Lipid metabolism: specifically, catabolism, biosynthesis, and peroxidation play critical roles in autophagy or apoptosis-mediated cellular homeostasis including cell survival and death (Xie et al., 2020). Autophagy can act as an anti-viral or pro-viral mechanism; however, most viruses are found to inhibit autophagy signaling (Jackson, 2015). In the case of SARS-CoV-2, we know little about the relationship of virus infection with autophagy signaling. Importantly, although we also observed elevated levels under normal growth conditions, we observed further elevation of the levels of ether lipids (plasmanyl and plasmenyl) with increased viral replication, which was reported as a key lipid class for efficient membrane trafficking (Thai et al., 2001). We revealed that lipids are critical molecular factors for SAR-CoV-2 infection, entry, and viral replication into host cells. These lipids, which were elevated by SARS-CoV-2 infection may be counteracted by PUFA and VLCFA that the virus suppresses upon infection and entry (Kang et al., 2019).

**Figure 8:**
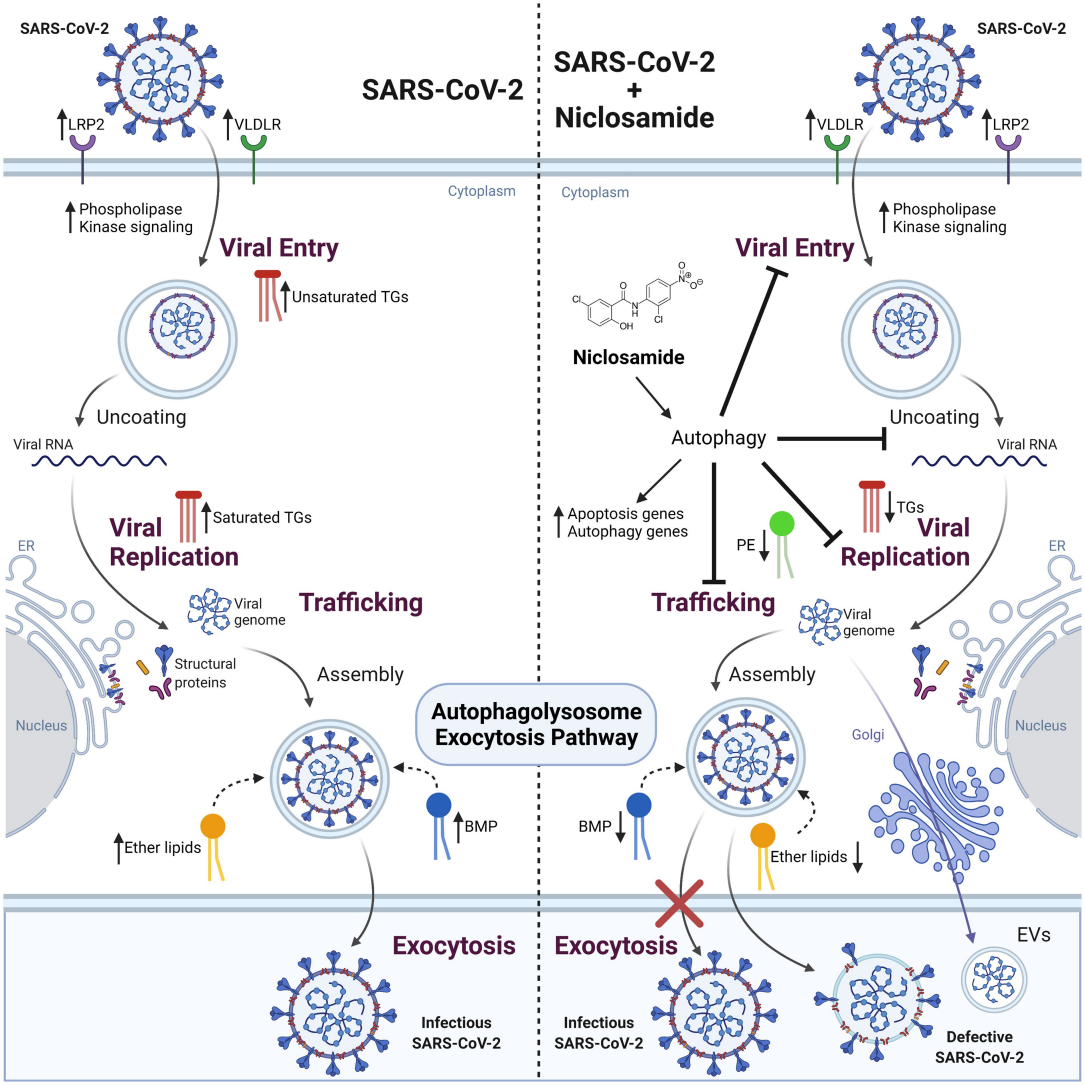
The effect of SARS-CoV-2 infection and NIC treatment on host cell lipid metabolism. SARS-CoV-2 infection in Vero E6 cells alters host cell lipid metabolism during early and late stages of infection. Increased transcription of lipid receptors LRP2 and VLDLR, and phosphorylation signaling regulators are observed throughout viral infection. Changes in TG composition from unsaturated to saturated acyl-chains occur as a function of viral replication, with an overall decrease in TG lipids at late infection timepoints. This change corresponds with an increase to DG and BMP lipids that is indicative of energy consumption and incorporation into membranes and vesicles, activation of autophagy pathways, as well as impacting viral replication. Treatment of cells with NIC alters lipid composition and gene regulation corresponding to apoptosis and autophagy related pathways. Decreases to ether lipids (TGs and DGs) and BMP are observed and reflect a decrease to exocytosis pathways for viral egress. Created with BioRender.com.

Collectively, these data provide new insight into the mechanism of action for the antiviral activity of NIC, namely induction of lipophagy through the induction of autophagic pathways (Figure 8), which expands as well as refines our understanding of the pleiotropic antiviral mechanisms described for NIC (Kao et al., 2018; Braga et al., 2021; Miao et al., 2021). We can mechanistically infer that SARS-CoV-2 infection wrests control of cellular autophagy to ensure a productive infection of the host cell. Therapeutically, we demonstrated that NIC treatment stimulated autophagy by elevating PE levels and in parallel, downregulated total ether lipids, TGs, and other lipid molecules. Reduction of these key lipids by NIC treatment may lead to inhibition of SARS-CoV-2-induced signaling, which in turn drives viral endocytosis, vesicular trafficking, propagation, and viral egress. Activating autophagy using small molecule inhibitors/activators, oxysterols, or peptides is a promising approach for treating viral infections (Rubinsztein et al., 2012; Chen and Zhang, 2022; Chen et al., 2023). However, most of the potential treatments (including NIC) suffer translational obstacles due to adverse side effects, inefficient drug delivery to target tissues, and poor bioavailability (Skotland et al., 2015). Although NIC is considered an essential medicine and is well tolerated, the long history of dosing safety is based on oral delivery and anti-parasitic activity within the gastrointestinal tract. SARS-CoV-2 infection begins in the lungs but has been shown in several clinical studies to have a cosmopolitan infectivity profile, including the brain, heart, and gastrointestinal tract. Notably, several repurposed drugs for COVID-19 were found to be associated with autophagy induction (Kim et al., 2012), raising the possibility that some drugs that are already in clinical use for the treatment of parasitic or bacterial infections may be acting, at least in part, via autophagy. The recent clinical testing of intranasal or inhaled NIC (Backer et al., 2021) suggests that the potential of this drug for use in mitigating COVID-19 severity is being explored in earnest.

Although we used a *C. sabaeus* kidney epithelial cell line (Vero E6) and a SARS-CoV-2 strain isolated from a Floridian patient, we observed a similar (in direction and magnitude) transcriptomic response to other SARS-CoV-2 transcriptomic studies using human cell lines, despite differences in MOI and experimental sampling timepoints. Furthermore, consistency in the lipidomic response detected between our initial and secondary experiments (conducted at an MOI of 0.001 and an estimated MOI of 0.5, respectively), suggests that the overall apoptotic and lipophagic responses are conserved in NIC and SARS-CoV-2 infections regardless of MOI. These changes were consistent across another Vero E6 study noting cell stress and apoptosis (DeDiego et al., 2011), as well as other cell systems, including human lung cells (Wyler et al., 2021), cardiomyocytes (Sharma et al., 2020), adenocarcinomic human alveolar basal epithelial cells (Daamen et al., 2021), and bronchial epithelial cells (Yoshikawa et al., 2010), infected with different betacoronaviruses, suggesting the responses detected herein may represent a core set of host SARS-CoV viral response genes, and that these genes are not exclusive to our study system. Although it is likely that Vero E6, a transformed cell line, have disrupted transcriptional regulation, lipidomes, lipid metabolism, lipophagic, and autophagic processes as compared to primary human cells, the phenomenological and phenotypic consistency reported above, supports the significance of the work reported herein. Furthermore, although our work is limited to an *in vitro* model, it was recently shown in a multiomics study of COVID-19 patient samples that the lipidomic profile can effectively partition COVID-19 disease severity (Xu et al., 2020); implying that the biology captured in our culture model is relevant *in vivo*. These data support thoughtful consideration of MOAs in screening for repurposed drugs and compels prioritized focused on drugs that promote the antiviral activities of lipophagy, which may in turn lead to the reduction of virus egress and the subsequent regulation of key lipid mediators of pathological inflammation.

## 4. Materials and methods

### 4.1. Data availability statement

The raw and processed data generated here have been deposited in publicly accessible databases; the RNA sequencing data is available through NCBI’s GEO repository (Accession: GSE178157), and the lipidomics data is accessible via MetaboLights (Accession: MTBLS5782). Any additional information required to reanalyze the data reported in this paper is available from the lead contact upon request.Vero E6 cell culture.

### 4.2. SARS-CoV-2 culture

Vero clone E6 cells (ATCC: CRL-1586) were obtained from Dr. Pei-Yong Shi (University of Texas Medical Branch). Vero E6 cells are an immortalized kidney epithelial cell line obtained from a female African green monkey (*Chlorocebus sabaeus*). These cells have been authenticated by a commercial testing laboratory and repository.

Cells were maintained at 37°C and 5% CO2, in Dulbecco’s Modified Eagle Medium (ThermoFisher #11965118) supplemented with 10% heat-inactivated fetal bovine serum (FBS), 1X L-glutamine (Gibco #25030–081), and 1X penicillin/streptomycin (Corning #30-001-CI). For all conditions, the cell culture was maintained at 3% FBS.

SARS-CoV-2/human/USA/UF-13/2020 (GenBank: MT620766.1) was passaged in Vero E6 cells, with culture conditions as described above except using reduced serum media with 3% FBS rather than 10% FBS during the initial infection and maintenance in reduced serum media until harvest. Virus was collected after 4 days to establish a low passage virus stock within our biosafety level 3 (BSL-3) laboratory at the Emerging Pathogens Institute.

### 4.3. Plaque assay

Vero E6 cells were seeded 48 h before they were infected at a density of 4,500 cells/well of a 96 well plate. A 10-fold dilution series of samples were applied to Vero E6 cells and incubated at 37°C with 5% CO2 for 1 h. After this 1-h incubation, an overlay of 0.8% methylcellulose prepared in reduced serum DMEM was added, and the cells were incubated at 37°C at 5% CO_2_ for 5 days, after which the overlay was removed, and cells were fixed and stained with 1% crystal violet (1, 1 methanol, acetone) for 30 min.

### 4.4. Experimental infection and niclosamide treatment

Cells were seeded in 6 well plates at a density of 500,000 cells/well. Each replicate sample represents an individual plate well, with three biological replicates per condition, per time point. The investigators were not blinded to the conditions. At hour zero (48 h post seeding), media was removed and replaced with reduced serum Dulbecco’s Modified Eagle Medium (supplemented with 3% FBS, 1X L-glutamine, and 1X penicillin/streptomycin) to facilitate infection.

One vial of SARS-CoV-2 stock was thawed and virus was inoculated into each well with 12.5 μL of diluted virus stock for an intended MOI of 0.001 infectious virus particles/Vero E6 cell, calculated from an estimated plaque forming units (PFU)/mL value based on the genome copy of the virus stock (1×10^11^ genome copies/mL). Subsequent experiments used a 1.5×10^6^ PFU/mL virus stock at a known MOI of 0.5.

Niclosamide [MedChemExpress #HY-B0497, (NIC)] stock was dissolved in molecular grade DMSO and added to the indicated wells for a final concentration of 5 μM (0.5% v/v DMSO final concentration). An equivalent amount of molecular grade DMSO was used as a vehicle control. This low concentration of NIC was selected to have antiviral effect but be well below the reported 50% cytotoxic concentration (CC_50_) of the drug in Vero E6 cells (250 μM) to avoid inadvertent cell lysis in treated conditions (Xu et al., 2020). In our culture system, no significant reduction in cell viability in comparison to vehicle control was seen at concentrations below 20 μM NIC (Supplementary Figures S2A,B).

Samples were prepared and collected following a time of addition experimental design (Aoki-Utsubo et al., 2018) as follows (Figure 1; Supplementary Table S1). The “0 h” (0 h) samples were collected 48 h after cell seeding. The “16 h” (16 h) conditions were infected with virus and treated with NIC or DMSO at hour zero and harvested at hour 16 of the experiment (Figure 1). The “48 h” (48 h) conditions were infected with virus at hour zero, supernatant was removed after 24 h, and cells were treated with NIC or DMSO in fresh media with 10% FBS. Cells were harvested at hour 48. Uninfected samples (with and without drug treatment) were also prepared and collected as above. For samples from infected conditions, the supernatant was removed at the indicated harvest timepoint, taking care not to disturb the cell monolayer, and retained at −80°C. The cells were washed once with 1X PBS. The PBS wash was removed and 0.25% trypsin–EDTA (ThermoFisher #25200056) was added to each well. The plates were then incubated at 37°C and 5% CO2 for 5–10 min until cells had completely detached from the cell surface. Trypsinization was halted by the addition of 1 mL FBS. The supernatant was removed, the cell pellet was resuspended in 1X PBS, and the sample was centrifuged at 700 RPM for 5 min. Supernatant was aspirated, and 100% methanol was added to each sample purposed for lipid analysis. Alternatively, 750 μL of TRIzol reagent (ThermoFisher #15596026) was added to each sample purposed for RNASeq. All samples were stored at −80°C until analysis. For uninfected conditions, harvest proceeded the same as described above except trypsinization was halted using 4 mL of complete Dulbecco’s Modified Eagle Medium instead of FBS, and pellets were flash frozen in liquid nitrogen instead of being resuspended in a chemical inactivation agent after the last centrifugation step. All samples were stored at −80°C until analysis.

### 4.5. Cell counting kit 8 (CCK-8) viability assay

Vero E6 cells were seeded in a 96 well plate at a density of 10,000 cells per well, with 3 technical replicate wells per condition. The experiment began at “0 h,” 48 h after seeding. For the dose curve experiment (Supplementary Figures S2A,B), at 0 h cells were transferred from complete DMEM to complete DMEM without phenol red (ThermoFisher #31053028) to avoid phenol red interfering with the absorbance measurement. For the experiment including virus infection (Supplementary Figures S2C,D), cells were transferred to reduced serum DMEM without phenol red. The 16 h samples were treated with the indicated concentration of NIC or DMSO vehicle control along-side virus at an MOI of 0.001 at 0 h and harvested at 16 h, and 48 h samples were infected at 0 h, treated at 24 h, and harvested at 48 h. The 10% DMSO condition functioned as a positive control expected to induce cell death. At the harvest timepoint, 10 μL of CCK-8 assay reagent (APExBIO #K1018) was added to each well, and cells were returned to the 37°C and 5% CO_2_ cell culture incubator for 90 min. The absorbance of the plate at 450 nm was determined using a Byonoy Absorbance 96 microplate reader.

### 4.6. Global lipid extraction

Cell pellets from each time point and condition were extracted using a modified Folch biphasic extraction procedure (Ulmer et al., 2018). Samples were pre-normalized to the protein concentration (800 μg/mL) obtained using a Qubit 4.0 Fluorometer (Thermo Fisher Scientific). Lipids were extracted using ice cold 4:2:1 chloroform:methanol:water (v:v:v) containing 20 μL of a 10X diluted internal standard mixture (stock solution of 50 ppm, w: v), and the organic phase was collected after centrifugation at 3260 *x g* for 5 min at 4°C, dried under nitrogen gas, and reconstituted in 75 μL of isopropanol (IPA) plus 1 μL of injection standard mixture (100 ppm, w,v). Internal standards used in this analysis covered a range of lipid classes and structures including: lysophosphatidylcholine (LPC 17:0), phosphatidylcholine (PC 17:0/17:0), phosphatidylethanolamine (PE 15:0/15:0), phosphatidylserine (PS 14:0/14:0), phosphatidylglycerol (PG 14:0/14:0), ceramide (Cer d18:1/17:0), diacylglycerol (DG 14:0/14:0), and sphingomyelin (SM d18:1/7:0). For injection standards, triacylglycerol (TG 17:0/17:0/17:0), LPC 19:0, PE 17:0/17:0, PS 17:0/17:0, and PG 17:0/17:0 were used. Except for TG, all other lipid standards were purchased from Avanti Polar Lipids (Alabaster, AL) while TG was purchased from Sigma Aldrich. All lipid standards were diluted prior to analysis in 1:2 (v/v) chloroform:methanol (CHCl3:MeOH) and a working standard mix was then prepared by diluting the stock solution with the same solvent mixture.

### 4.7. LC-HRMS-based lipid data collection and analysis

Ultra-high-pressure liquid chromatography coupled to high resolution mass spectrometry (UHPLC-HRMS) was used for data collection. Chromatographic separation was achieved using reversed phase chromatography (Dionex Ultimate 3,000 RS UHLPC system, Thermo Scientific) with a Waters Acquity C18 BEH column (2.1 × 50 mm, 1.7 μm) (Waters, Milford, MA, United States). The mobile phases consisted of solvent A (60:40 acetonitrile:water) and solvent B (90:8:2 isopropanol:acetonitrile:water), both containing 10 mM ammonium formate and 0.1% formic acid. The flow rate was 500 μL/min and the column temperature was maintained at 50°C. A multi-step gradient was used for separation starting with 0% B from 0–1 min, increasing to 30% B from 1–3 min, then up to 45% B from 3–4 min, 60% B from 4–6 min, 65% B from 6–8 min, held at 65% B from 8–10 min, increased to 90% B from 10–15 min, then increased to 98% B from 15–17 min and finally held at 98% B from 17–18 min before returning to initial conditions to equilibrate for the next injection. Samples were analyzed in positive and negative electrospray ionization as separate injections on a Thermo Scientific Q-Exactive high resolution mass spectrometer (Thermo Scientific, San Jose, CA). Lipidomics data were compiled and annotated using LipidMatch (Koelmel et al., 2017).

### 4.8. RNA extraction

For total RNA extraction from flash frozen cells, the cell pellet was thawed on ice. Cell pellets were resuspended in 1 mL TRIzol, and 200 μL chloroform was added to each sample. Samples were mixed thoroughly by vortexing, then incubated at room temperature for 3 min. Samples were centrifuged at 12,000 *x g* for 15 min at 4°C. The upper aqueous layer was transferred to a new tube and 500 μL isopropanol was added and mixed by vortexing, prior to incubation at room temperature for 10 min. RNA was pelleted by centrifuging at 12,000 *x g* for 10 min at 4°C, and supernatant was discarded. The pellet was rinsed in 75% ethanol and pelleted by centrifuging at 7,500 *x g* for 5 min at 4°C. The ethanol wash and spin were repeated a second time. The pellet was air dried in an inverted tube. Genomic DNA was digest-ed using the TURBO DNA-free™ Kit (Invitrogen #AM1907) according to manufacturer instructions. Samples were mixed with 350 μL buffer RLT from the RNeasy® Mini kit (Qiagen #74104) and 250 μL 100% ethanol, then transferred to a RNeasy® Mini kit spin column. Columns were centrifuged at 8,000 *x g* for 15 s at room temperature, and flow-through was discarded. Buffer RPE (500 μL) was added to the column, which was centrifuged at the same conditions, and flow-through was discarded. The RPE wash was repeated a second time. The column was transferred to a fresh collection tube and centrifuged at 12,000 *x g* for 1 min to dry. RNA was eluted by adding 50 μL of nuclease free water and centrifuging for 1 min at 8,000 *x g* and was immediately frozen at −80°C. To extract viral RNA from culture supernatant, supernatant was thawed, 200 μL of supernatant was mixed with 200 μL DNA/RNA Shield, and RNA was immediately extracted using the Quick-DNA/RNA™ Viral MagBead kit (Zymo Re-search #R2140), according to manufacturer instructions and then immediately frozen at −80°C.

### 4.9. Real-time RT-qPCR

To quantify the virus genome copies in supernatant, one step real time reverse transcription qPCR (RT-qPCR) was performed using 4x Quantabio UltraPlex 1-Step ToughMix No Rox (VWR #10804–946) and CDC 2019-nCoV_N1 (nucleocapsid) primer and probe mix (see Key Re-sources Table for primer and probe sequences) at a final concentration of 22.5 μM, in a 20 μL reaction volume with 5 μL template. All samples were run in technical duplicates. RT-qPCR was run on a BioRad CFX96™ Real-Time System. The thermal cycling conditions were as follows: 50°C for 20 min, 94°C for 2 min; followed by 45 cycles of 94°C for 15 s, 55°C for 30 s, and 68°C for 10 s. The samples were quantified using a standard curve generated using a 2019-nCoV_N_Positive Control plasmid (IDT # 10006625). Standard curve points were plotted in Microsoft Excel v.2102 and cycle threshold values of unknown samples were determined from the logarithmic line of best fit equation to calculate genome copies/mL.

### 4.10. RNASeq analysis

All 27 RNA samples (see Supplementary Table S1) were directly sent to Novogene,^1^ where in-house RNA quality control and library preparation was performed. Libraries were sequenced on an Illumina NovaSeq6000 platform using a 150 bp kit with paired end read mode. RNA sequencing was only performed on the initial experiment.

### 4.11. Mapping, expression, and pathway, analyses

Bioinformatic processing was completed using Galaxy^2^ (Ulmer et al., 2018). Quality control was performed with Cutadapt v 1.16 (Martin, 2011) and FastQC v 0.11.8^3^ to remove adapters, <20 nucleo-tide reads and low-quality reads. RNA STAR v2.7.7a (Batut et al., 2018) was used to map samples to the *Chlorocebus sabaeus* genome and associated annotation (GenBank accession # GCA_015252025.1). featureCounts v 2.0.1 (Liao et al., 2014) was used with Infer Experiment v2.6.4.1 (Wang et al., 2012) to determine the strandedness of the samples, and sum reads for each gene. Read counts for each sample were input into DESeq2 v 1.22.1 (Love et al., 2014) to call differential gene expression, analysing the effect of time, drug, and virus on the samples. Resultant differential expression files were manually annotated using a combination of NCBI (O'Leary et al., 2016) and Ensembl (Yates et al., 2020) due to the poor annotation quality of the genome.^4^ Significantly regulated gene names were separately parsed out for each comparison and uploaded to ENRICHR (Kuleshov et al., 2016) for downstream gene ontology GO (Harris et al., 2004) and pathway analyses using Reactome (Fabregat et al., 2016), MSigDB (Liberzon et al., 2015), and KEGG (Ogata et al., 1999) databases.

### 4.12. Statistical analysis

Unless indicated in the figure legend, all experiments were performed in triplicate and results are presented as mean ± standard error of mean (SEM) of absolute values or percentages of control. Each replicate was a separate tissue culture well processed in parallel. A list of all samples used for subsequent analysis can be seen in Supplementary Table S1.

Statistical *p*-values were obtained by application of the appropriate statistical tests using GraphPad Prism v.9.0. Lipidomics data was normalized to the total ion signal, glog transformed, autoscaled and analyzed with Metaboanalyst v.3.0 (Pang et al., 2021). For quality control, internal lipid standards added to each sample were used to assess technical reproducibility, achieving ≤15% relative standard deviation (RSD) across all samples. Correlation heatmaps were generated for the lipid samples using Pearson’s r. Heatmap clustering was performed using a *T*-test or an ANOVA, showing the most significant lipids. For lipidomics applications, Bonferroni false discovery rate (FDR) adjusted p-values lower than 0.05 and an absolute fold-change greater than or equal to 1.5 were considered significant. For RNASeq differential gene ex-pression, gene ontology and pathway analyses, statistical significance was corrected for multiple comparisons (Bonferroni adjusted) and assessed at an *α* = 0.05. Genes with absolute log2 fold changes ≥1 were considered significantly differentially expressed. For supernatant genome copy RT-qPCR, CCK-8, and plaque assay data, a two-way non-parametric ANOVA with Dunn’s post-hoc test was performed in GraphPad Prism v.6.0 and assessed at an *α* = 0.05. Since no statistical differences were observed between technical replicates of the same condition in the supernatant genome copy RT-qPCR data, technical replicate wells were pooled in the reported analysis.

## Data availability statement

The raw and processed data generated here have been deposited in publicly accessible databases; the RNA sequencing data is available through NCBI’s GEO repository (Accession: GSE178157), and the lipidomics data is accessible via https://zenodo.org/record/8381788. Any additional information required to reanalyze the data reported in this paper is available from the lead corresponding author upon request.

## Author contributions

RD, TG, and JL conceptualized the study. IM, HC, TH, CS, MM, and JA conducted the study and generated samples and data. IM, HC, TH, MM, JA, HY, RD, and TG analyzed the data. IM, HC, TH, MM, JA, JL, RD, and TG wrote and edited the manuscript. All authors contributed to the article and approved the submitted version.

## Footnotes

^1^https://en.novogene.com/

^2^https://galaxyproject.org

^3^http://www.bioinformatics.babraham.ac.uk/projects/fastqc/

^4^https://www.ncbi.nlm.nih.gov/genome/annotation_euk/Chlorocebus_sabaeus/100/
