# Supplementary material for: Niclosamide as a chemical probe for analyzing SARS-CoV-2 modulation of host cell lipid metabolism
Source: Front Microbiol. 2023 Oct 11;14:1251065. doi: 10.3389/fmicb.2023.1251065 (PMC10603251; doi:10.3389/fmicb.2023.1251065)
Supplement: Supplementary file 1 [file Data_Sheet_1.zip › Supplementary figures.pdf]

SUPPLEMENTARY FIGURES

A chemical probe into SARS-CoV-2 modulation of host cell lipid metabolism

Timothy J. Garrett[1,2*], Heather G. Coatsworth[3‡], Iqbal Mahmud[1,2‡], Timothy Hamerly[3‡], Caroline J. Stephenson[3,4], Hoda S. Yazd[5], Jasmine Ayers[3], Megan Miller[3], John A. Lednicky[3,4], Rhoel R. Dinglasan[3*]

[1]Department of Pathology, Immunology, and Laboratory Medicine, College of Medicine, University of Florida, Gainesville, FL, 32610 USA
[2]Southeast Center for Integrated Metabolomics, Clinical and Translational Science Institute, University of Florida, Gainesville, FL, 32610 USA
[3]Emerging Pathogens Institute, Department of Infectious Diseases and Immunology, College of Veterinary Medicine, University of Florida, Gainesville, FL, 32611 USA
[4]Department of Environmental and Global Health, College of Public Health and Health Professions, University of Florida, Gainesville, FL, 32610 USA
[5]Department of Chemistry, University of Florida, Gainesville, FL 32603 USA

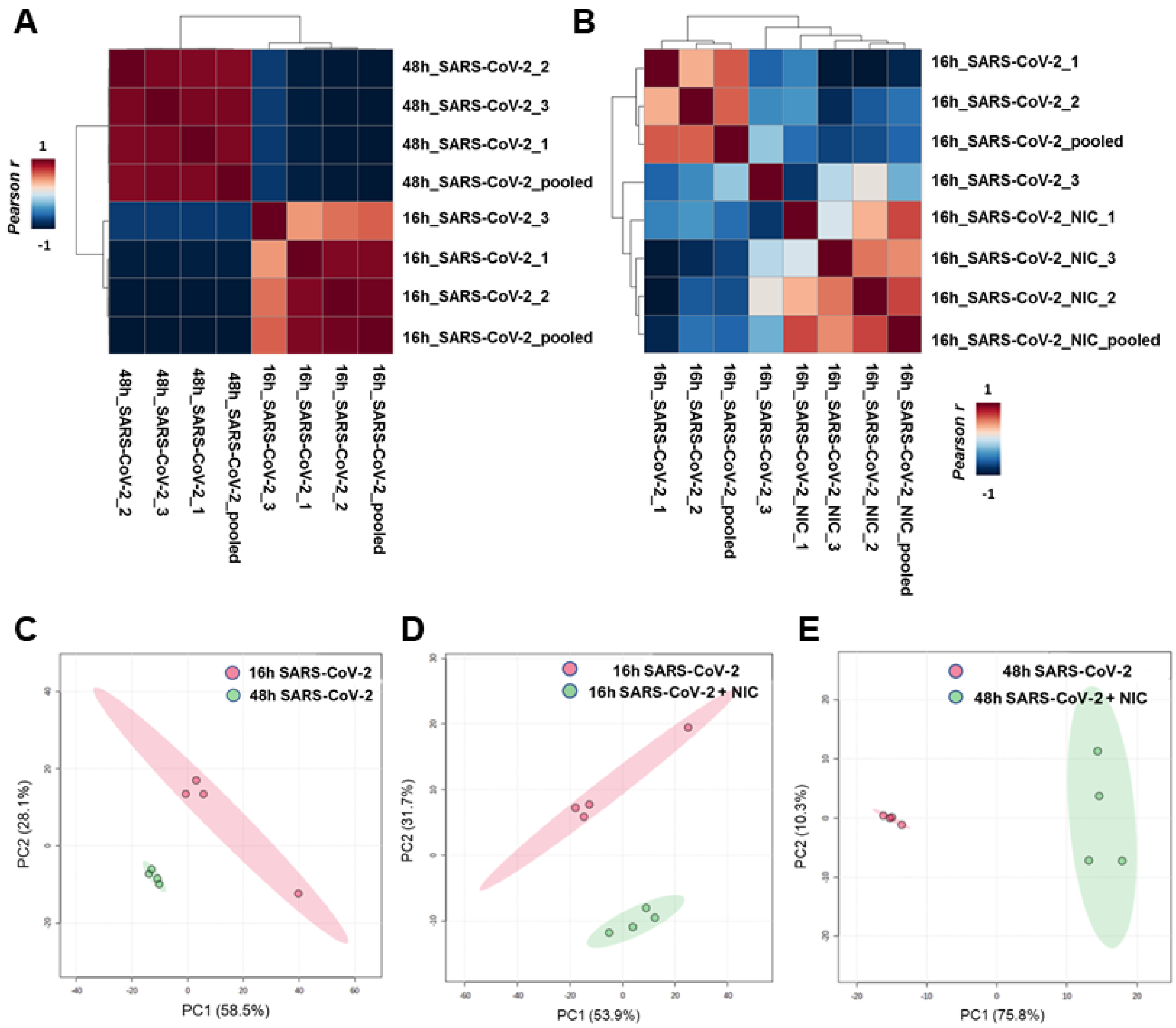

Figure S1. Pearson correlation analyses on lipidomic data. (A) Viral infection at early (16h) and late time points (48h). **(B)** Viral infection with NIC treatment at 16h. Principal components analysis (PCA) of early and late viral infection **(C)** and with NIC treatment at early (16h) and late (48h) time points **(D-E)**. Clustering was evident with viral infection and with treatment using Pearson correlation and PCA. A pooled sample from each group is identified and was used to evaluate clustering. Data representative of intended MOI of 0.5.

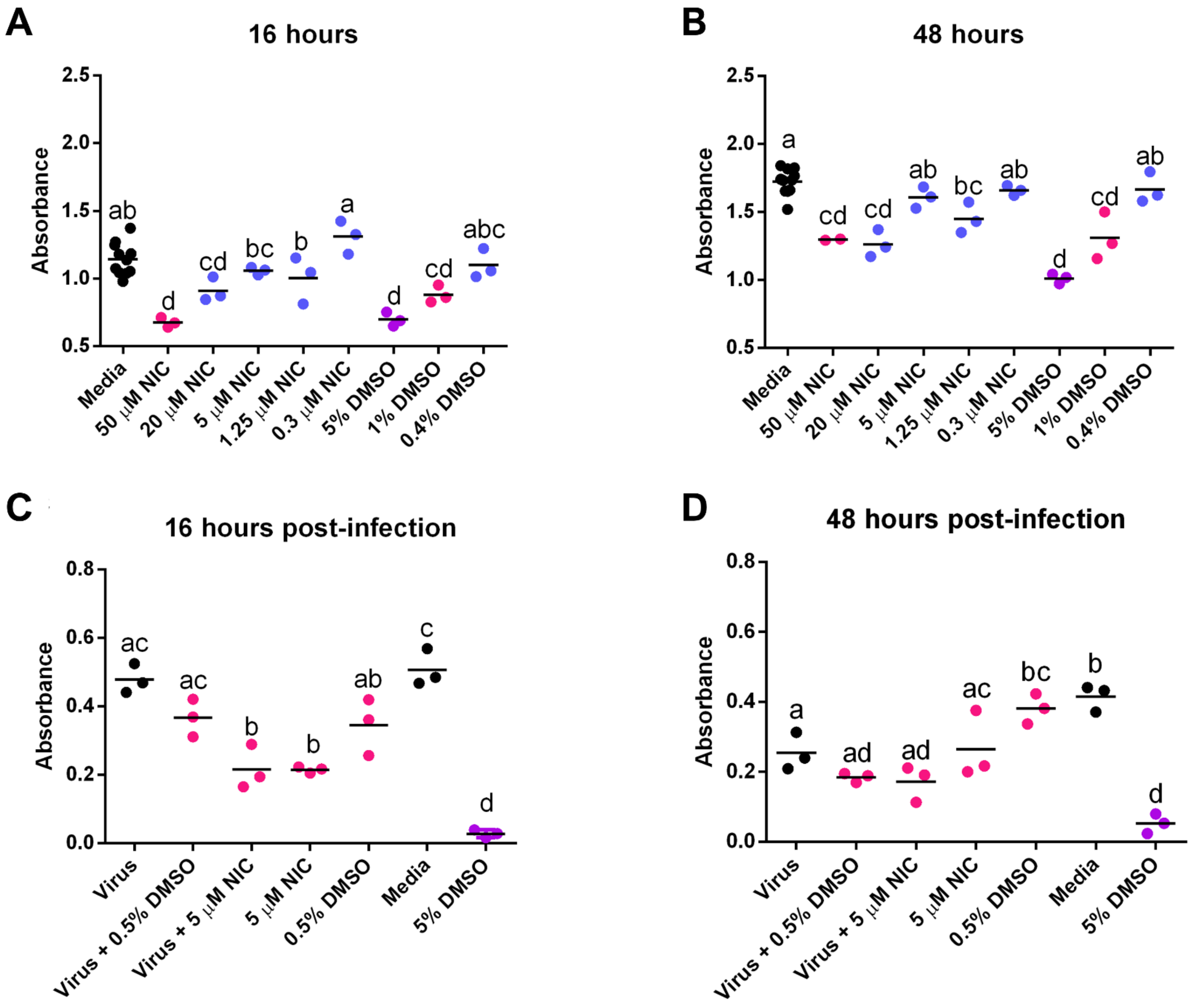

Figure S2. Viability of NIC and virus treated Vero E6 cells. (A-B) Cytotoxicity dose curve of NIC and DMSO vehicle in complete media at 16 h post-treatment **(A)** and 24 hours post-treatment (48h post initiation of experiment) **(B)**. Conditions with black points contain no DMSO, blue contains 0.4% DMSO, pink contains 1% DMSO, and purple contains 10% DMSO. **(C-D)** Effect of DMSO, Niclosamide, and virus infection on viability in Vero E6 cells in reduced serum media at 16h post-treatment and infection **(C)** or 48h post-infection, 24h post-treatment **(D)**. Conditions with black points contain no DMSO, pink contains 0.5% DMSO, and purple contains 5% DMSO. Values reported are absorbance at 450 nm 1.5 hours after addition of 10 μl CCK-8 assay reagent per well of a 96 well plate. Each point represents a technical replicate culture treated simultaneously. Conditions that do not share a letter were found to be statistically significantly different, $\alpha < 0.05$, by two-way non-parametric ANOVA with Dunn's test for post-hoc pairwise comparisons.

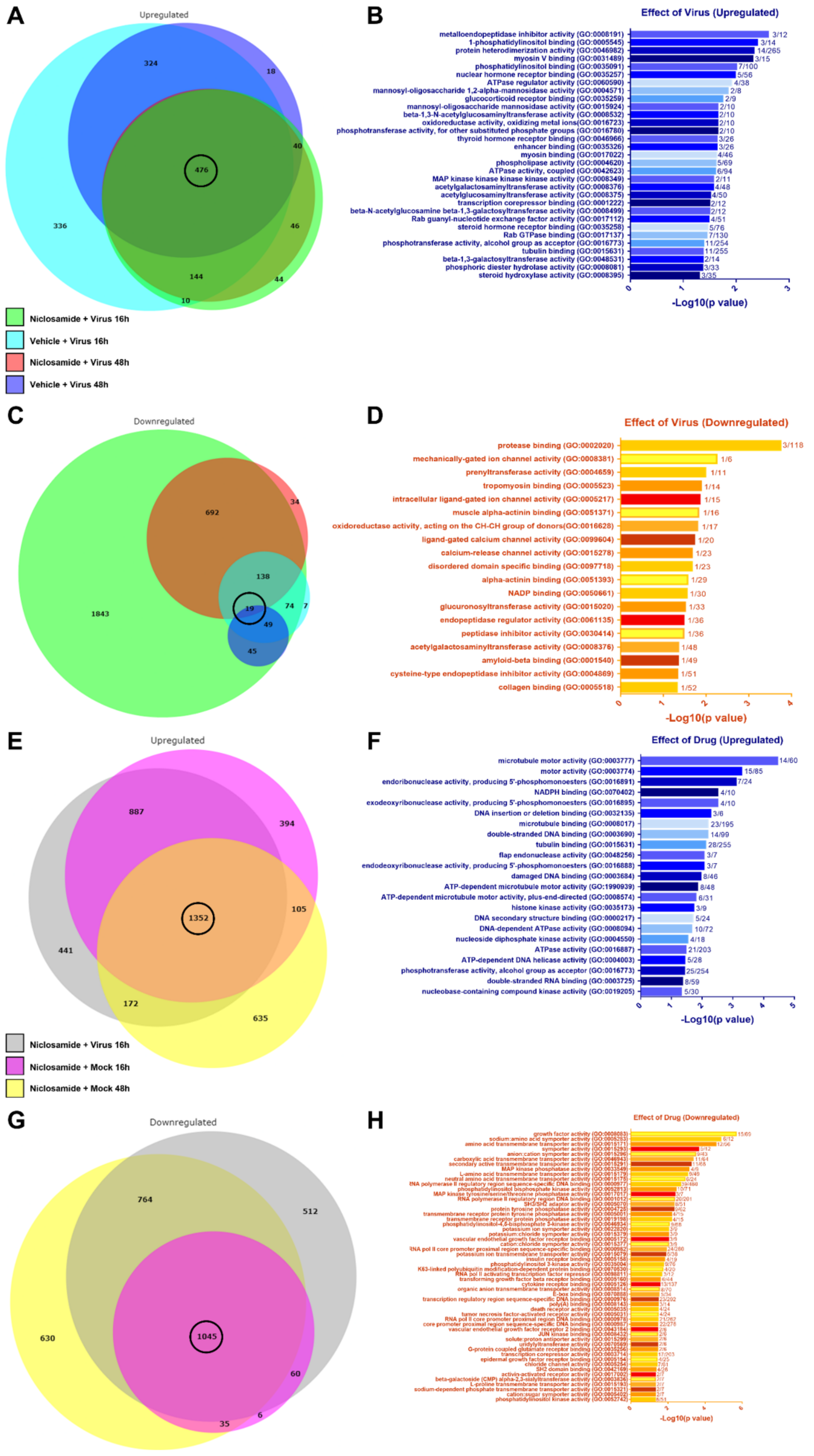

Figure S3. The number of overlapping differentially expressed genes and statistically significant overrepresented gene ontology (GO) terms depicting the transcriptomic effect of SARS-CoV-2 infection and niclosamide treatment. (A,C) Venn diagram demonstrating the number of shared differentially expressed genes by different virus-specific conditions, (A)

Upregulated genes and (C) Downregulated genes. **(B,D)** Virus samples (with niclosamide at 16 and 48 hours, and with vehicle [DMSO] at 16 and 48 hours) were analyzed against no-virus samples and combined to analyze global virus specific transcriptome changes. Bar labels represent the number of differentially regulated genes as a function of the total number of possible genes within the listed GO term. (B) Significantly upregulated genes, colored in blue, (D) Significantly downregulated genes, colored in red and yellow. **(E,G)** Venn diagram demonstrating the number of shared differentially expressed genes by different niclosamide-specific conditions, (E) Upregulated genes and (G) Downregulated genes. **(F,H)** Niclosamide samples (with mock at 16 and 48 hours, and virus at 16 hours) were analyzed against no-drug treated samples and combined to analyze global drug specific transcriptome changes. Bar labels represent the number of differentially regulated genes as a function of the total number of possible genes within the listed GO term. (F) Significantly upregulated genes, colored in blue, (H) Significantly downregulated genes, colored in red and yellow. Genes were designated as significantly differentially expressed using DESeq2 when their Bonferroni adjusted P-value was ≤ 0.05, and the $|\log2(\text{Fold Change})|$ was ≥ 1. GO statistical significance was performed using ENRICHR and was adjusted for multiple comparisons and tested at $\alpha = 0.05$.

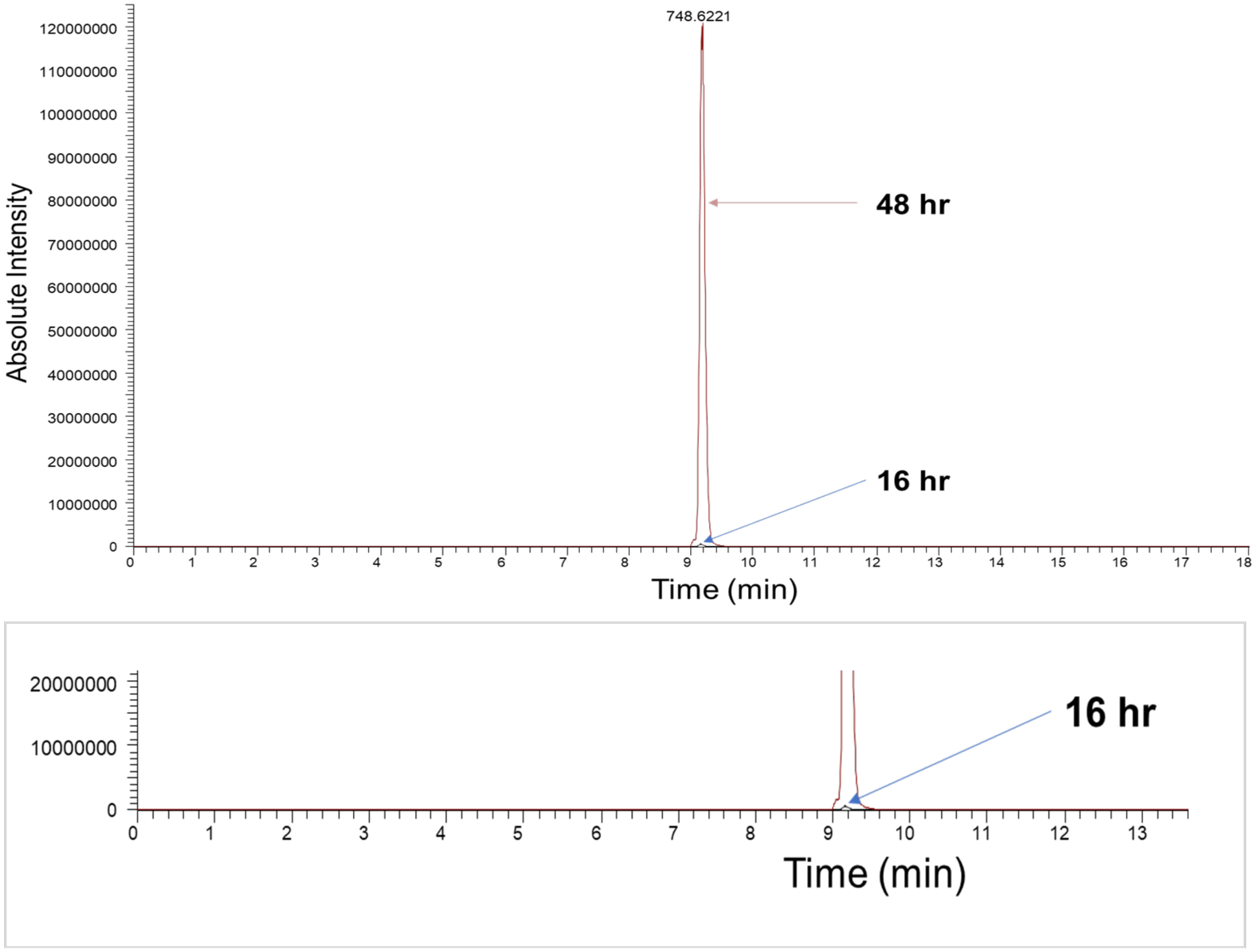

Figure S4. A comparative extracted ion chromatogram for plasmanyl-PC (O-18:0/16:0)+H) at 16h vs. 48h following SARS-CoV-2 infection. (Top) The relative peak intensity of this single ether lipid between 16h (blue) and 48h (dark red) infection. The arrows points to the lipid identified as Plasmanyl-PC (O-18:0/16:0)+H at retention time 9.16. (Bottom) Enlarged image showing the small peak at 16h, demonstrating clearly differentiated profiles. Identification was confirmed by accurate mass and MS/MS.
